# Functional duplication of Rap1 in methylotrophic yeasts

**DOI:** 10.1038/s41598-019-43595-8

**Published:** 2019-05-10

**Authors:** Alexander N. Malyavko, Olga A. Petrova, Maria I. Zvereva, Olga A. Dontsova

**Affiliations:** 1 Faculty of Chemistry and Belozersky Institute of Physico-Chemical Biology, Lomonosov Moscow State University, Moscow, 119992 Russia; 2 Center of Life Sciences, Skolkovo Institute of Science and Technology, Moscow, 143026 Russia; 3 Shemyakin-Ovchinnikov Institute of Bioorganic Chemistry of the Russian Academy of Sciences, Moscow, 117997 Russia

**Keywords:** Telomeres, Transcriptional regulatory elements

## Abstract

The telomere regulator and transcription factor Rap1 is the only telomere protein conserved in yeasts and mammals. Its functional repertoire in budding yeasts is a particularly interesting field for investigation, given the high evolutionary diversity of this group of unicellular organisms. In the methylotrophic thermotolerant species *Hansenula polymorpha* DL-1 the RAP1 gene is duplicated (HpRAP1A and HpRAP1B). Here, we report the functional characterization of the two paralogues from *H*. *polymorpha* DL-1. We uncover distinct (but overlapping) DNA binding preferences of HpRap1A and HpRap1B proteins. We show that only HpRap1B is able to recognize telomeric DNA directly and to protect it from excessive recombination, whereas HpRap1A is associated with subtelomere regions. Furthermore, we identify specific binding sites for both HpRap1A and HpRap1B within promoters of a large number of ribosomal protein genes (RPGs), implicating Rap1 in the control of the RP regulon in *H*. *polymorpha*. Our bioinformatic analysis suggests that RAP1 was duplicated early in the evolution of the “methylotrophs” clade, and the two genes evolved independently. Therefore, our characterization of Rap1 paralogues in *H*. *polymorpha* may be relevant to other “methylotrophs”, yielding valuable insights into the evolution of budding yeasts.

## Introduction

The termini of eukaryotic chromosomes – telomeres – are organized differently compared to its internal regions. Telomeric DNA consists of multiple short G/C-rich sequences that serve as binding sites for telomere-specific proteins. The formation of distinct nucleoprotein structures at the ends of the chromosomes is necessary for the protection of genomic DNA. Due to the end-replication problem, telomeres shorten after each cell division and eventually lose their protective functions^1^. This problem can be solved by telomerase – an enzyme with the ability to synthesize telomeric repeats, using its own RNA template within the RNA component of telomerase (TER). Single-cell eukaryotes (like budding yeast) with a disrupted TER gene are able to undergo a limited number of cell divisions, and upon reaching the critical telomere length, they enter a replicative senescent state and most cells die^2^. However, several survivors can resume telomere maintenance via recombination-based mechanisms^3^. Both telomerase action and recombination are controlled by proteins that specifically bind telomeric DNA.

Repressor-activator protein 1 (Rap1) – first discovered as a factor recognizing a conserved element in the promoters of a number of translational apparatus genes^4^ (including most of the ribosomal protein genes (RPG)) – is an essential transcription factor that controls the expression of hundreds of important genes, a mating-type loci silencer and a major component of telomere chromatin in *Saccharomyces cerevisiae*^5–7^. The high-affinity GGTGTGTGGGTGT telomere site is specifically recognized by a duplicated Myb domain^8^, with each Myb domain contacting one of the 5-bp GGTGT half-sites^9^. However, ScRap1 can accommodate many nucleotide substitutions in these half-sites (with or without a loss in binding affinity) by altering the side chain conformations of DNA-contacting residues^10^, making it a very versatile DNA-binding protein. This property is reflected in a set of loosely defined ScRap1 recognition motifs determined by several high-throughput methods (e.g., KRTGTRYGGGTGT)^11^. Perhaps such DNA binding promiscuity is one of the reasons for the high sequence divergence of telomeric repeats in other budding yeast species^12^.

Near the N-terminus, Rap1 contains a BRCT domain that interacts with Gcr1 factor, and this interaction is necessary for the regulation of glycolytic genes transcription^13^. In addition, Rap1 BRCT has been implicated in the maintenance of cell wall homeostasis^14^. The Rap1 C-terminal (RCT) domain plays a crucial role at *S*. *cerevisiae* telomeres, serving as a platform for loading of Sir3/Sir4 and Rif1/Rif2 pairs of proteins. While the former is required for establishing subtelomere silencing^15^, Rif1/Rif2 are largely involved in the regulation of telomeric DNA turnover^16,17^. Rif1 and Rif2 inhibit telomerase recruitment at telomeres^18,19^ and protect telomeres from C-strand resection^20,21^ via only partially overlapping pathways. In addition, the RCT domain (through Sir4 and Rif2) and central DNA-binding domain of Rap1 ensure protection from telomere fusions via a non-homologous end joining (NHEJ) mechanism^22^.

Rap1 is the only telomere protein that is conserved from yeast to human. However, in fission yeast and mammals, Rap1 apparently lacks the ability to bind telomeric DNA directly, which is attributed to the presence of only one copy of the Myb domain in its DBD. Instead, its localization to chromosome ends relies on the interaction of the RCT domain with Taz1 and TRF2 (direct dsDNA binders in *Schizosaccharomyces pombe* and mammals, respectively)^23,24^. Despite different telomere recruitment modes, SpRap1 shares many functions with its budding yeast counterpart – it is necessary for telomerase regulation, protection from telomere fusion and transcriptional control^25–27^. In higher eukaryotes, the involvement of Rap1 in telomere maintenance is somewhat controversial: while several reports implicate mammalian Rap1 in length regulation and NHEJ suppression^24,28–30^, gene knockouts both in human cell lines and mice do not confirm these findings^31,32^. Mammalian Rap1, however, does appear to be required for protection against homology-directed repair (HDR)^31,33^. Interestingly, both in mice and humans, Rap1 has been found to be involved in transcription regulation outside telomeres, and the conservation of Rap1 has been proposed be due to gene expression control^32,34^.

**Figure 1:**
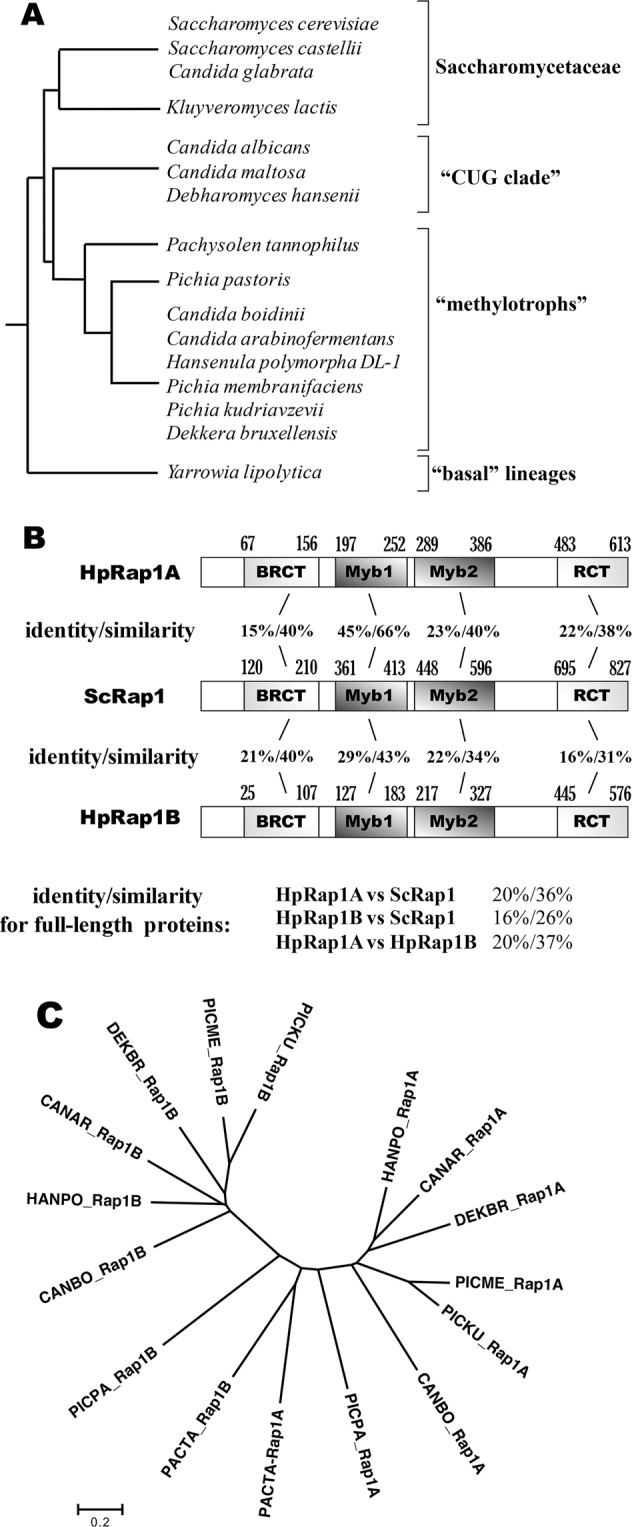
Rap1 paralogues in methylotrophic yeasts. (**A**) The four major groups of *Saccharomycotina* as defined in^35^. Tree topology based on^73^. (**B**) Percentage of identity/similarity between amino acid sequences of the indicated Rap1 homologues. (**C**) Phylogenetic relationships of the Rap1 homologues from methylotrophic yeasts were inferred using the Neighbor-Joining method. The optimal tree with the sum of the branch length = 7.44450490 is shown. The tree is drawn to scale, with branch lengths in the same units as the evolutionary distances used to infer the phylogenetic tree. Species abbreviations are as described in “METHODS”.

Not only have Rap1 homologues from evolutionarily distant organisms functionally diverged, but several significant changes can be observed even in more closely related budding yeasts (subphylum *Saccharomycotina*). Based on the available genomic data, budding yeasts can be divided in four subgroups with distinct genome organizational features (Fig. 1A)^35^. Each of these groups apparently experienced major changes in the requirement for a particular Rap1 function during evolution. *Yarrowia lipolytica* (a representative of the “basal” lineages of the *Saccharomycotina*) lacks the RAP1 gene, and its most prominent functions (RP gene and telomere regulation) are performed by other proteins. Telomeres of *Y*. *lipolytica* are bound and protected by Tay1 protein^36^, whereas the Tbf1 transcription factor has been predicted to bind *Y*. *lipolytica* RPG promoters^37^. The Rap1 homologue from the human pathogen *Candida albicans* (a representative of the “CUG clade”) lacks the important RCT domain. CaRap1 binds telomeric repeats and controls telomere maintenance via recombination, but it apparently lacks the ability to inhibit telomerase action^38^. Moreover, in *C*. *albicans* (and most likely other members of the “CUG clade”), Rap1 binding sites are lost from RP gene promoters, which are instead regulated by Tbf1 and Cbf1 factors^37,39^. Rap1 homologues from members of the *Saccharomycetaceae* family of budding yeasts appear to be similar to *S*. *cerevisiae*. Rap1s from *Saccharomyces castellii* and *Kluyveromyces lactis* are able to regulate telomerase action, and at least KlRap1 requires the RCT domain for this function^40,41^. The RCT domain of Rap1 from *Candida glabrata* and *K*. *lactis* has been implicated in subtelomere silencing^42,43^. In addition, *K*. *lactis* mutant telomeres that are defective in Rap1 binding are highly recombinogenic^44^. Finally, Rap1 recognition motifs can be found in RP gene promoters of yeasts from the *Saccharomycetaceae* family^45^.

In the present manuscript, we report that members of the “methylotrophs” clade – the fourth large subgroup of the *Saccharomycotina* – contain a duplicated RAP1 gene. Using the thermotolerant methylotrophic yeast *Hansenula polymorpha* as a model, we show that two Rap1 homologues have different preferences for DNA binding. Due to this divergence, only one of the Rap1s is able to specifically recognize telomeric repeats in *H*. *polymorpha* (and perhaps in other “methylotrophs”) and suppress telomere truncations via recombination. Moreover, using a combination of *in silico*, *in vitro* and *in vivo* methods, we show that both Rap1 homologues can bind promoters of RP genes, suggesting their involvement in transcriptional control.

## Methods

### Yeast strains and constructs

The strains used in this study are listed in Supplementary Table S1. Oligonucleotides used for PCR during strain construction are listed in Supplementary Table S2. The DL1-L strain^46^ was used as a wild type (no tag) control in all experiments. The diploid *H*. *polymorpha* CBS4732 strain was obtained by crossing haploid 1B^47^ and 1B27^48^ strains on maltose-containing medium (2% maltose, 3% agar) and selecting for Ade^+^Ura^+^ prototrophy. Gene replacements were performed by transformation of the DLdaduA strain^49^ (or another strain where indicated) with DNA integration cassettes according to a standard protocol, modified as described^47^. Templates for PCR amplification of integration cassettes were obtained by subcloning (detailed in Supplementary methods). Correct integration of the cassettes and gene replacements were verified by PCR. The absence of unwanted mutations was verified by sequencing.

Doubling times of yeast strains were determined as follows. Four single colonies of the indicated yeast strain were grown overnight in YPD medium (1% yeast extract, 2% peptone and 2% D-glucose) at 37 °C. Overnight cultures were diluted into fresh YPD medium to OD_600_ ~0.05 and grown at 37 or 47 °C. The OD_600_ was measured each hour for 8 hours, and the obtained values were fitted into the “Exponential growth equation” (GraphPad Prism version 7.00). Means and standard deviation between four replicate doubling times were then calculated.

### Bioinformatic procedures and protein expression and purification

Described in Supplementary methods.

### Chromatin Immunoprecipitation (ChIP)

Yeast cells were grown to OD_600_ ~0.9 in 50 ml of YPD at 37 °C and fixed with 1% formaldehyde for 10 min at 25 °C. Cross-linking was stopped by addition of glycine (125 mM final concentration) and incubation for 5 min at 25 °C. Cells were harvested by centrifugation, washed with TBS (50 mM Tris, pH 7.6; 150 mM NaCl), resuspended in 0.5 ml of lysis buffer A (50 mM HEPES pH 7.5, 140 mM NaCl, 1 mM EDTA, 0.1% Triton X-100, 0.01% sodium deoxycholate and Halt Protease and Phosphatase Inhibitor Cocktail (Thermo Scientific)) and broken with glass beads in a Precellys Evolution homogenizer. After addition of 0.5 ml of lysis buffer B (50 mM HEPES pH 7.5, 140 mM NaCl, 1 mM EDTA, 1.9% Triton X-100, 0.19% sodium deoxycholate and Halt Protease and Phosphatase Inhibitor Cocktail (Thermo Scientific)), the cells were sonicated to shear the chromatin (300–500 bp median fragment size), and the cell debris was removed by centrifugation (14000 g, 15 min). Next, 0.75 ml of lysates were incubated with 20 µl of Pierce anti-HA magnetic beads (Thermo Scientific) at +4 °C for 3 hours. Beads were washed once with 1 ml of lysis buffer (50 mM HEPES pH 7.5, 140 mM NaCl, 1 mM EDTA, 1% Triton X-100, 0.1% sodium deoxycholate), once with 1 ml of high-salt lysis buffer (50 mM HEPES pH 7.5, 1 M NaCl, 1 mM EDTA, 1% Triton X-100, 0.1% sodium deoxycholate), twice with 1 ml of wash buffer (10 mM Tris pH 8, 1 mM EDTA, 0.25 M LiCl, 0.5% NP-40, 0.5% sodium deoxycholate, 0.05% SDS) and once with 1 ml of TE (10 mM Tris pH 8, 1 mM EDTA). Chromatin was eluted in two steps: first with 250 μl of 0.1 M NaHCO_3_, 1% SDS by incubation at 65 °C for 15 min, followed by incubation with 250 μl of 0.1 M NaHCO_3_, 1% SDS by incubation at room temperature for 10 min. The two fractions were pooled, supplemented with 0.2 M NaCl and incubated at 65 °C overnight to reverse cross-linking. Subsequently, 20 μl of 1 M Tris pH 6.5 and 10 μl of 0.5 M EDTA were added along with 30 μg of RNase A and incubated at 37 °C for 30 min. The samples were then treated with 100 μg of proteinase K at 37 °C for 1 hour. DNA was extracted with phenol/chloroform, precipitated with EtOH and dissolved in 25 μl of TE buffer. Similarly, DNA was isolated from 7.5 μl of the lysates (1% of the input chromatin) and used for normalization. Real-time PCR was performed in a 15 μl mixture containing 0.45 μl of DNA (either “IP” or “1% of input” sample), 1x Taq KCl buffer, 2.5 mM MgCl_2_, 0.6 μM primers, 0.2 mM dNTPs, 0.4x SYBR Green and 0.9 U of Taq polymerase (Thermo Fisher Scientific). The primers used in ChIP are listed in Supplementary Table S3. We used the standard deviation between four replicates (cultures from four colonies of the same strain) as a value of the experimental error. For statistical analysis two-way ANOVA followed by Dunnett’s multiple comparisons test was performed using GraphPad Prism version 7.00.

### EMSA (Electrophoretic mobility shift assay)

Oligonucleotide sequences are listed in Supplementary Table S3. To prepare the double-stranded FAM-HANPO2 DNA substrate, 50 μl of a mixture containing FAM-HANPO2s and FAM-HANPO2as (10 μM each) in PBST buffer was incubated at 98 °C for 2 min and slowly (2 °C/min) cooled to room temperature. Double-stranded FAM-TEL and FAM-TEL1 were obtained by PCR with TELf/TELr and TELf/TEL1r primer pairs using yeast genomic DNA as template, dATP/dGTP/dCTP (0,2 mM each), 0.17 mM dTTP, 0.033 mM FAM-dUTP (Thermo Fisher Scientific) and the Q5 polymerase (NEB) kit according to the manufacturer’s instructions. PCR products were then purified using the “Cleanup Mini” kit (Evrogen).

Reactions (20 μl) were performed in a solution of 20 mM HEPES-NaOH, pH 7.5, 50 mM NaCl, 0.2 mg/ml bovine serum albumin, 5% glycerol and 0.1 mg/ml of salmon sperm DNA (ACROS Organics). In the competition assay, non-labeled oligonucleotides were added immediately after the addition of protein. The mixtures were incubated for 15 min at room temperature, followed by the addition of 1 μl of loading buffer (0.5x TBE, 50% glycerol, bromophenol blue, xylene cyanol). Next, 5 μl of the reactions were loaded on a 4% non-denaturing polyacrylamide gel (19:1). Electrophoresis was performed at room temperature at 175 V for 20 min.

### Surface plasmon resonance (SPR) DNA binding assay

The ProteOn XPR36 (Bio-Rad) system was employed for all SPR experiments. The oligonucleotide sequences are listed in Supplementary Table S3. For reusable DNA immobilization, we utilized an approach similar to the ReDCaT technique^50^. 2 nM (in PBST buffer) biotinylated single-stranded DNA oligonucleotide (bio-linker) was injected at a flow rate of 30 mkl/min for 60 s over the neutravidin-coated surface (NLC sensor chip) in all ligand flow channels to provide a relatively low immobilization level of ~10 RU. All further experiments were conducted in SPR buffer (10 mM HEPES-NaOH, pH 7.5, 150 mM NaCl, 0.5 mM DTT, 0.05% Tween 20, BSA 1 mg/ml, salmon sperm DNA 0.02 mg/ml). Test dsDNA substrates (containing single-stranded 3′-overhang complementary to the bio-linker; pre-annealed as described for the FAM-HANPO2 oligonucleotide) were diluted to 125 nM in SPR buffer and injected over the “bio-linker”- conjugated NLC surface at a 25 mkl/min flow rate for 60 s in ligand flow channels (typically resulting in an ~15–30 RU immobilization level). After three SPR buffer injections (100 mkl/min flow rate, 60 s) into the analyte flow channels, the protein was injected at a 75 mkl/min flow rate for 100–350 s, followed by a dissociation phase (100–600 s). Two or three replicate protein injections over the same interaction spot were performed in each experiment. In addition, blank buffer was injected in a parallel channel to correct for baseline drift. Signal from “interspots” – surface free of any DNA – served as a control for nonspecific protein interactions with the empty surface and “bulk effect”. For high-affinity interactions, protein was removed by injection of 1 M NaCl, 0.1% CHAPS (100 mkl/min flow rate, 20 s); in case of low-affinity substrates, all protein molecules were dissociated by the end of a dissociation phase without the need for regeneration. Test DNAs were removed by injection of 50 mM NaOH (100 mkl/min flow rate, 30 s). After double referencing (blank buffer reference and interspot reference), the experimental data were globally fitted to a Langmuir 1:1 kinetic model with mass transfer. Additionally, ${{\rm{K}}}_{{\rm{d}}}^{{\rm{app}}({\rm{eq}})}$ were derived from the equilibrium analysis (in cases where equilibrium was reached for all protein concentrations tested). Kinetic and equilibrium parameters were determined in at least three independent experiments and reported as the mean ± SD in Supplementary Table S4 (full report) and Table 1 (only ${{\rm{K}}}_{{\rm{d}}}^{{\rm{app}}}$ values calculated from kinetic fits).

**Table 1:** Affinities of the interaction of the DNA oligonucleotides with HpRap1 paralogues.

Oligo name	sequence 5′-3′ (only sense strand is shown)	Rap1A		Rap1B	
		${{\bf{K}}}_{{\bf{d}}}^{{\bf{app}}}$, nM	performance in competition assay	${{\bf{K}}}_{{\bf{d}}}^{{\bf{app}}}$, nM	performance in competition assay
VIIrAbs*	GGCAGGCGGTGTAGGGATGCGGC	0.28 ± 0.09	+++	64 ± 2	−
CANAL^#^	ACTTCTTGGTGTACGGATGTCTA	0.050 ± 0.014	+++	9.1 ± 0.7	+
CANMA^#^	CTCGCTTGGTGTACGGATGCAGA	nd	+++	nd	+
DEBHA^†^	GTGTTGAGGTGTAGGGATGTTGA	nd	+++	nd	+
SACCE^#^	GTGTGTGGGTGTGTGGGTGTGTG	39 ± 15	+	3.6 ± 0.4	++
HANPO2^#^	GGGTGGCGGGGTGGCG	NB	nd	12 ± 1	nd
HANPO4^#^	GGGTGGCGGGGTGGCGGGGTGGCGGGGTGGCG	NB	−	12 ± 3	+
PICKU^†^	TGAACTAGGAGCGAGGTGTGTTAC	NB	−	0.27 ± 0.08	+++
CANAR^†^	GGTGTTGGGTGTTGGGTGTTGGG	NB	−	3.1 ± 0.4	++
PACTA^†^	TGTGAGTGGTGTGAGTGGTGTGAGTGG	nd	−	nd	+
PICPA^†^	GGATGCTGGATGCTGGATGCTGGATGCT	nd	−	nd	−
PICME^†^	TAGAACTAGGTGTGCGGGGTATAATAG	nd	−	nd	++
DEKBR^†^	GGATTGGTGGATTGGTGGATTGGT	nd	−	nd	−
RPS16^‡^	CAAACCCCGTGTAAGGGTGCCTA	0.50 ± 0.08	nd	NB	nd
RPS0a^‡^	AGAGGGCTGTGCACGGATGCTAT	10 ± 4	nd	NB	nd
RPL5a^‡^	CTTGTGTGGTGCAGGGGTGGCAG	1.4 ± 0.3	nd	2.0 ± 0.0	nd
RPS14^‡^	GTGTTCAGGTGTGAGGTGTGTTT	NB	nd	0.048 ± 0.007	nd
RPS0b^‡^	ATTTCTTGGTGCGAGGGGGCTCT	NB	nd	1.5 ± 0.4	nd
RPL36b^‡^	GGACAGTGGTGTAGGGGGTAGAG	NB	nd	2.5 ± 0.2	nd
RPS22b^‡^	AATGTTCGGTTCGAGGAGTTCTT	NB	nd	2.8 ± 0.6	nd
rnd1	ACGACTCACTGTAGATACGACTCACTGTAGAT	NB	−	NB	−
rnd2	AAATCTAGACATGAAAAAAAAAATGTTAGTAATCGAAATCTC	nd	−	nd	−

“NB” - no binding; “nd” - not determined. *Subtelomere HpRap1A binding site; ^#^known telomeric repeats from *C*. *albicans*^74^, *C*. *maltosa*^75^, *S*. *cerevisiae*^76^, and *H*. *polymorpha*^52^; ^†^putative telomeric sequences extracted from the genome sequences of *D*. *hansenii*^77^ and yeasts from the “methylotrophs” clade (Supplementary Table S5); ^‡^HpRap1 recognition sequences from RPG promoters. Underlined regions mark the 12-13-bp core Rap1 recognition sequences that we deduced based on the crystallographic studies of ScRap1^10^, binding experiments with CaRap1^38^ and results of this study.

For the competition SPR assay, we immobilized either VIIrAbs (for HpRap1A) or PICKU (for HpRap1B) dsDNA as described above and injected 2 nM protein preincubated in SPR buffer (for 1 h) with either 20 nM (“10x”) or 200 nM (“100x”) of the competitor dsDNA (without the 3′-overhang complementary to the bio-linker) at a 75 mkl/min flow rate for 200 s. The surface between protein injections was regenerated by 1 M NaCl, 0.1% CHAPS (100 mkl/min flow rate, 18 s). After the data processing (interspot referencing), responses at the end (160–200 s) of the association phase were extracted and divided to the response from a “no competitor” control.

### Western blots

Total proteins were isolated from 2.5 ml of YPD cultures (OD_600_) as previously described. Then, 6 µl of the protein fractions were loaded on a 10% denaturing polyacrylamide gel, and the proteins were transferred to a Hybond P 0.45 PVDF membrane (GE Healthcare) and stained with Ponceau S (Amresco). The membrane was blocked in 5% BSA and then incubated with 200 ng/ml rat anti-HA monoclonal (clone 3F10) antibodies (Sigma-Aldrich) and 2 µg/ml goat anti-rat antibodies (conjugated with Alexa Fluor 555, Thermo Fisher Scientific).

### Telomere southern blots

Southern blot experiments were conducted as previously described^51^ with one modification: we used 5′-radiolabeled C4 oligonucleotide 5′-(CGCCACCC)_4_-3′ as a probe. At least two isolated transformants were passaged and analyzed for each mutant strain. The cells were streaked onto a YPD plate and grown at 37 °C for 2 days (1^st^ streak). Then, a smear of cells from the first streak was restreaked on a second YPD plate and grown at 37 °C for another 2 days (2^nd^ streak). Cells after the indicated number of streaks were grown overnight in 10 ml of YPD and used for genomic DNA isolation. Assuming that each streak involved approximately 20 cell divisions (and accounting for ~30 cell divisions during transformation and analysis), cells from the 4^th^ and 7^th^ streaks underwent approximately 110 and 170 population doublings, respectively.

### Equipment and settings

The Coomassie-stained protein gel image was captured using the ChemiDoc™ XRS System (Bio-Rad) using the “White epi illumination” application (no contrast adjustments were applied). The fluorescence images from EMSA experiments were acquired on the Typhoon FLA 7000 (GE Healthcare) imaging system using the “FAM” method 473 nm (with no contrast adjustments). The fluorescent Western blot images were acquired on the ChemiDoc™ MP System (Bio-Rad) using the “Alexa 546” application; band intensities were determined using ImageLab 5.2.1 software (no contrast adjustments were applied). The Ponceau S-stained membranes were captured using the ChemiDoc™ XRS System (Bio-Rad) with the “White epi illumination” (left panel) or “White Transillumination” (right panel) application (with no contrast adjustments). The Southern blot images were acquired on the Typhoon FLA 7000 (GE Healthcare) imaging system using the “Phosphor” method. Minor contrast adjustments were performed in ImageQuant TL 7.0 and applied equally across the entire image (including controls).

## Results

### Rap1 paralogues in *H*. *polymorpha* and related species

Blast search against the *H*. *polymorpha* protein database using the sequence of *S*. *cerevisiae* Rap1 as a query yielded two open reading frames: HpRap1A (UniProt accession number W1Q7Z6) and HpRap1B (UniProt accession number W1QDN2). HpRap1A is slightly more similar to ScRap1 (20% identity) than HpRap1B (16% identity with ScRap1) (Fig. 1B). Substantial similarity between ScRap1 and its *H*. *polymorpha* homologues was revealed in all three domains (BRCT, duplicated MYB and RCT); the DNA-binding MYB domains displayed the highest percentage of identity (Figs 1B and S1). HpRap1A and HpRap1B were almost as different from each other as any of them from ScRap1, and corresponding genes were located on different chromosomes, suggesting their early divergence. We were able to identify two Rap1 homologues in eight sequenced genomes of the yeasts from the “methylotrophs” clade, including *Pachysolen tannophilus* – the basal species within the clade. Multiple alignment and phylogenetic analysis indicated that the identified ORFs represent two families of proteins that evolved independently (Fig. 1C). The only exception was two Rap1s from *P*. *tannophilus* that were less different from each other, and for which assignment to either the A- or B-subfamily was ambiguous (Fig. 1C). We could not find a second B-like Rap1 in any other *Saccharomycotina*. Thus, we propose that a duplication of the RAP1 gene occurred in the last common ancestor of “methylotrophs”, with subsequent subfunctionalization of the two genes (with the exception perhaps of *P*. *tannophilus*).

### Telomeric repeats are bound by Rap1B, whereas Rap1A is a subtelomere protein in *H*. *polymorpha*

**Figure 2:**
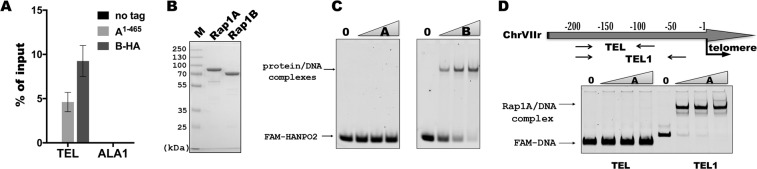
HpRap1B is a telomere protein, and HpRap1A is a subtelomere protein. (**A**) ChIP analysis. Chromatin from the indicated strains was immunoprecipitated on anti-HA magnetic beads. DNA was analyzed by qPCR with primers targeting either subtelomere region of the right end of chromosome VII, approximately 50 bp from the first telomeric repeat (“TEL”) or ALA1 gene locus (negative control), located approximately 100 kb from the right end of chromosome VII (“ALA1”). The amount of DNA fragment in the IP samples is represented as a percentage of the input DNA (mean ± SD); four replicates (cultures from four colonies of each strain) were used in the experiment. (B) Aliquots of purified HpRap1 proteins were analyzed by SDS-PAGE and Coomassie staining. Expected protein size of HpRap1A – 69.2 kDa, HpRap1B – 66.8 kDa. HpRap1A runs in the gel with lower than expected mobility, and its identity was confirmed by mass spectrometry. (**C**) EMSA: 50 nM FAM-HANPO2 oligo was incubated with an increasing amount of either HpRap1A (left panel) or HpRap1B (right panel) protein (concentration range: 0, 50, 100, 250 nM). The images were cropped from different gels and delineated with black dividing lines and white space. Full-length gels are shown in Supplementary Fig. S5. (**D**) Schematic of the regions of the right end of chromosome VII used in EMSA (upper panel). +1 – is the first base pair of the first telomeric repeat. “TEL” here is the same fragment as in the ChIP assay. (**A**) EMSA (lower panel): 10 nM FAM-labeled genomic fragments (“TEL” or “TEL1”) were incubated with an increasing amount of HpRap1A (0, 10, 25, 50 nM). The image was cropped from a single gel. The full-length gel is shown in Supplementary Fig. S6.

To examine whether the identified homologues are present at *H*. *polymorpha* telomeres, we created a strain expressing HpRap1B protein tagged with the hemagglutinin epitope (HA) at the C-terminus (B-HA). We could not obtain a strain with tagged full-length HpRap1A, presumably because the tag interferes with the protein function, and HpRap1A is essential for cell viability (like its homologue in *S*. *cerevisiae*). Instead, we constructed a strain expressing the 1–465 fragment of HpRap1A (without the RCT domain) tagged with HA epitope at its C-terminus (A^1–465^). Chromatin immunoprecipitation (ChIP) experiments revealed that, following immunoprecipitation on anti-HA beads, DNA fragments containing the telomere proximal region (“TEL”) (but not the control internal region “ALA1”) co-eluted with both HpRap1A and HpRap1B (Fig. 2A). Thus, both Rap1 paralogues localize at chromosome ends *in vivo* in *H*. *polymorpha*.

To verify whether the observed HpRap1 telomere localization was due to direct recognition of telomeric repeats, we tested their ability to bind telomeric DNA *in vitro* (*H*. *polymorpha* telomeric repeat unit is 5′-GGGTGGCG-3′)^52^. We expressed both proteins in *E*. *coli* and performed EMSA (Electrophoretic Mobility Shift Assay) using purified recombinant proteins and FAM-labeled telomeric oligonucleotides. Surprisingly, only Rap1B (but not Rap1A) shifted the 16-bp oligonucleotide with two 5′-GGGTGGCG-3′ repeats (HANPO2, Fig. 2B,C).

These observations contradict our ChIP results showing that both proteins bound near chromosomal ends *in vivo*. One possibility is that Rap1A has a binding site within subtelomere regions rather than telomeres. We tested this idea by performing EMSA using fragments from −1 to −200 bp of the chromosome VIIr subtelomere as probes. Indeed, we found that one DNA fragment (slightly longer than the one we used for PCR in the ChIP experiment) was very efficiently bound by Rap1A (Fig. 2D). Competition mobility shift experiments with shorter oligonucleotides revealed a minimum high-affinity Rap1A binding site (VIIrAbs) localized within the region from −76 to −54 bp of the chromosome VIIr subtelomere (Supplementary Fig. S2). Remarkably, VIIrAbs contains a 13-bp sequence, which is almost identical to the Rap1 binding site within the telomeric repeat from *C*. *albicans*^38^ (Table 1). The exact 13-bp sequence of VIIrAbs is also located very close to the telomere at the right end of the chromosome IV. Since VIIr and IVr are the only subtelomeres with telomeric sequences in the available assembly of the *H*. *polymorpha* DL-1 genome, we could not check whether the Rap1A binding sequence is present at all subtelomeres. However, we found the 13-bp sequence of VIIrAbs at the left end of the chromosome I. Moreover, three sequences of *H*. *polymorpha* DL-1 chromosomal ends, which were cloned in a study by Sohn *et al*.^52^ and could not be unambiguously assigned to any of the assembled chromosomes, also contained the Rap1A site. Thus, Rap1A can be loaded at least at 6 (out of 14) subtelomeres of *H*. *polymorpha* DL-1.

### Distinct DNA-binding specificities of Rap1 paralogues

Results from the previous section indicate that Rap1A and Rap1B may have different DNA recognition properties. We decided to further explore this possibility by testing the ability of HpRap1 proteins to interact with DNA oligonucleotides containing telomeric repeats from different budding yeasts. In addition to known telomeric repeats from *S*. *cerevisiae* and *C*. *albicans*, we utilized several putative telomeric repeats from other “methylotrophs”. We extracted putative telomeric (short terminal G/C-rich) repeats from seven of the available genomes (Tables 1 and S5).

**Figure 3:**
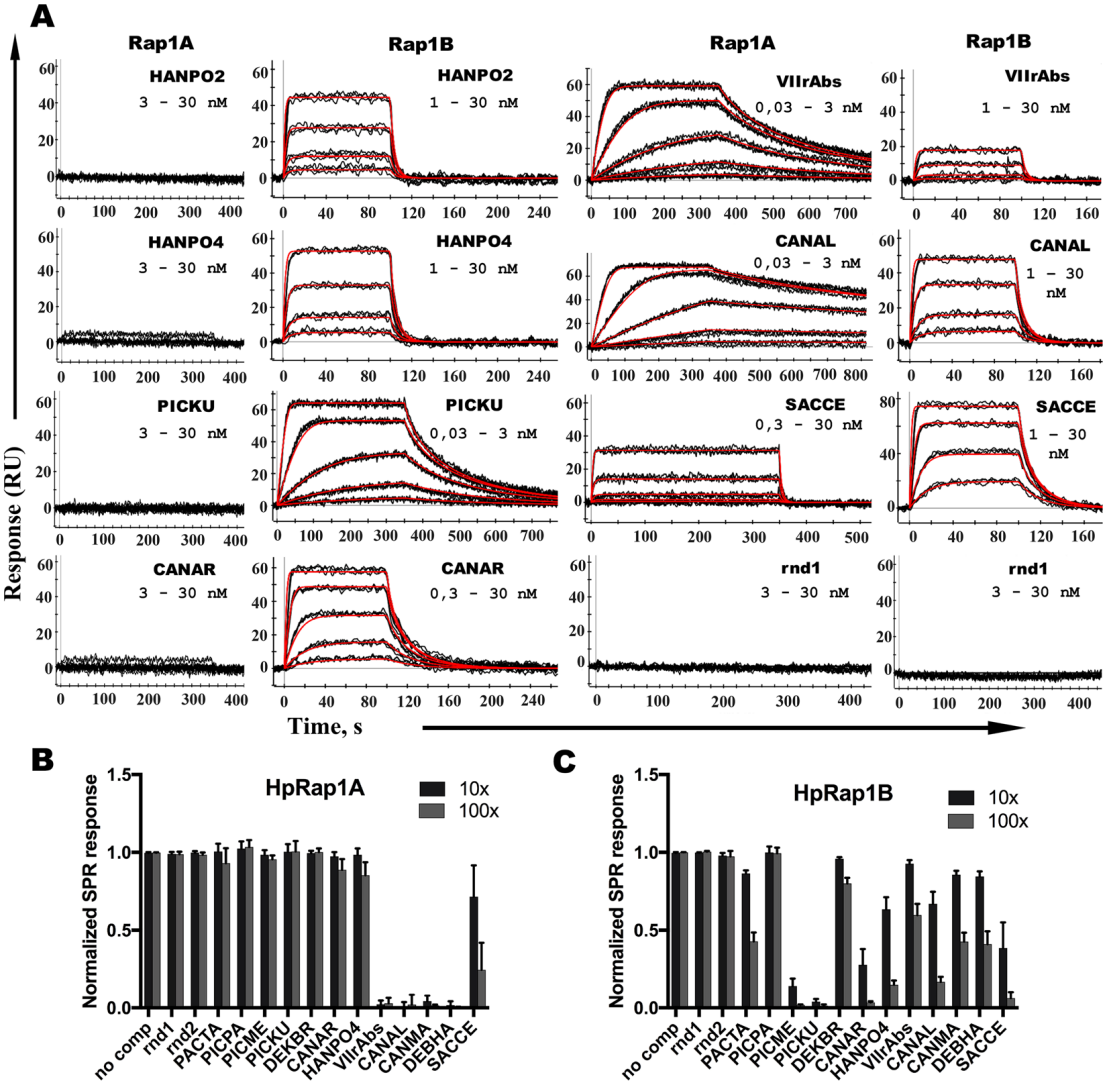
Distinct binding preferences of HpRap1 paralogues towards telomeric DNA from budding yeasts. (**A**) Sensograms showing the kinetic responses of HpRap1A (“Rap1A”) or HpRap1B (“Rap1B”) binding to the indicated DNA oligonucleotides (refer to Table 1 for sequences). The protein concentration range used in each case is indicated (0,03–30 nM means 0,03; 0,1; 0,3; 1; 3; 10 and 30 nM). Experimental data are shown in black, and the fits into the Langmuir 1:1 kinetic model with mass transfer are shown in red. K_d_ values are shown in Table 1, and the full kinetic report is presented in Supplementary Table S4. Three independent experiments were performed to obtain each K_d_. (**B**) HpRap1A binding specificity determined by the competition SPR experiment. The 2 nM HpRap1A preincubated with either 20 nM (“10x”) or 200 nM (“100x”) of the indicated oligonucleotide was injected over the VIIrAbs-containing surface (“no comp” – no competitor DNA control). Responses at the end of the injection phase were measured and normalized to the “no comp” control. Data from three independent experiments are plotted in the diagram (mean ± SD). (**C**) Same as (**B**) but with HpRap1B protein and the PICKU-containing surface.

To quantitatively measure HpRap1 affinities towards different DNA substrates, we used the surface plasmon resonance (SPR) assay. The results of the SPR assay confirmed the EMSA experiments: HpRap1B specifically interacted with HANPO2 and HANPO4 oligonucleotides with moderate affinity (${{\rm{K}}}_{{\rm{d}}}^{{\rm{app}}}$ = 12 ± 1 nM and 12 ± 3 nM, respectively); HpRap1A did not interact with HANPO2 and bound HANPO4 with at least 30-fold lower efficiency than HpRap1B (Fig. 3A, Table 1). Neither protein recognized an unrelated sequence of similar length (rnd1 oligonucleotide, Fig. 3A, Table 1). Remarkably, HpRap1B (but not HpRap1A) exhibited substantially higher affinity towards putative telomeric repeats from *Candida arabinofermentans* and *Pichia kudriavzevii* than to its cognate 5′-GGGTGGCG-3′ repeats (${{\rm{K}}}_{{\rm{d}}}^{{\rm{app}}}$ = 3.1 ± 0.4 nM for CANAR and 0.27 ± 0.08 nM for PICKU oligonucleotides, Fig. 3A, Table 1).

The marked difference between HpRap1B and HpRap1A responses in the SPR assay most likely is not due to poor HpRap1A preparation, since (consistent with the EMSA experiments, Fig. 2D) HpRap1A robustly binds the subtelomere VIIrAbs sequence (${{\rm{K}}}_{{\rm{d}}}^{{\rm{app}}}$ = 0.28 ± 0.09 nM) and *C*. *albicans* telomeric DNA with even higher affinity (0.050 ± 0.014 nM). Albeit much weaker, HpRap1A can also interact with the major telomeric repeat unit of *S*. *cerevisiae* (${{\rm{K}}}_{{\rm{d}}}^{{\rm{app}}}$ = 39 ± 15 nM) (Fig. 3A, Table 1). Interestingly, HpRap1B cannot discriminate well between *C*. *albicans*, *S*. *cerevisiae* and *H*. *polymorpha* telomeres (as well as putative telomeres from several other “methylotrophs”), since it binds them with low nanomolar ${{\rm{K}}}_{{\rm{d}}}^{{\rm{app}}}$ (Fig. 3A, Table 1).

To confirm that the differences obtained in affinities were not artifacts of the DNA immobilization, we performed SPR competition experiments. We also analyzed more putative telomeric oligonucleotides from the “methylotrophs” and “CUG” clades. Indeed, we found that none of the putative telomeric repeats of the “methylotrophs” showed any substantial interaction with HpRap1A, whereas five (of seven tested) bound HpRap1B to varying degrees (Fig. 3B,C, Table 1). In contrast, both HpRap1A and HpRap1B interacted with telomeres from the “CUG” clade and *S*. *cerevisiae*. Thus, we conclude that HpRap1 paralogues have distinct yet overlapping DNA binding properties.

### HpRap1B controls telomere length

Our attempts to generate strains with deletions of either HpRap1 paralogue were unsuccessful, which may indicate that both genes are essential for viability. To test this possibility, we analyzed the viability of meiotic progeny of strains heterozygous for either HpRAP1A or HpRAP1B. Since this analysis is not feasible in *H*. *polymorpha* DL-1 (due to its semi-sterility)^53^, we used its closest sibling *H*. *polymorpha* CBS4732 (a.k.a. *Ogataea polymorpha*). We disrupted one copy of either HpRAP1A or HpRAP1B with the LEU2 marker gene in diploid *H*. *polymorpha* CBS4732. Then (after sporulation) we performed random spore analysis by diethyl ether killing. None of the haploid segregants (of the 96 tested; 48 for each gene) produced any colonies on medium without leucine (Supplementary Fig. S3A,B), suggesting that disruption of either HpRAP1 paralogue is detrimental for growth. We also analyzed twenty (10 for each gene) random spores by PCR and observed only wild type alleles (Supplementary Fig. S3C,D). Thus, we conclude that both RAP1A and RAP1B are essential for cell viability in *H*. *polymorpha*.

**Figure 4:**
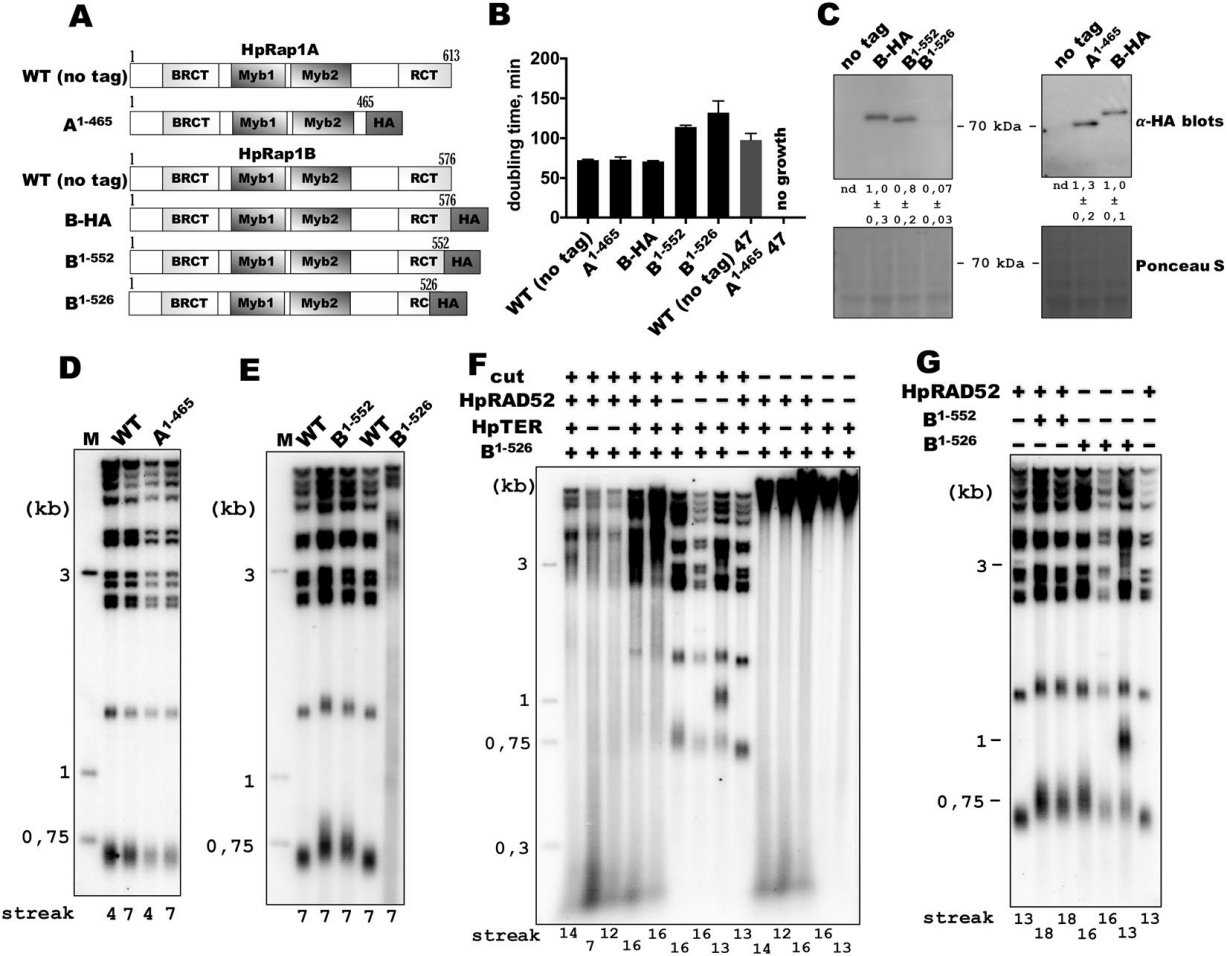
Phenotypes of the *H*. *polymorpha* strains with C-terminal truncations of Rap1 paralogues. (**A**) Schematic illustration of the mutant HpRap1A or HpRap1B proteins expressed in the indicated strains. “HA” – hemagglutinin tag. (**B**) Doubling times of the mutant strains grown in liquid YPD at 37 °C or 47 °C (mean ± SD between four replicate values). (**C**) Western blot analysis of the total proteins isolated from the indicated strains using the HA epitope antibody. Ponceau S-stained membranes served as a loading controls. The position of the 70 kDa marker band is shown. The band intensities were normalized to the signal from the B-HA strain. Data are represented as the mean ± SD calculated using four replicate values (proteins from four colonies of each strain). “nd” – not determined. The images were cropped from different membranes and are delineated by black dividing lines and white space. Full-length blots are presented in Supplementary Fig. S8. (**D**–**G**) Southern blot analysis of terminal restriction fragments from the indicated HpRap1 mutant strains. Genomic DNA was isolated from the strains after the N^th^ streak (where N is a number under a lane; each streak is ~20 generations). “M” – lane with telomeric DNA containing fragments that served as markers of length (their sizes are indicated on the left of each blot). (**F**) and (**G**) The B^1–526^ mutation was introduced (“+”) or not (“−”); the B^1–552^ mutation was introduced (“+”) or not (“−”); the HpTER gene is present in the genome (“+”) or knocked out (“−”); the HpRAD52 gene is present in the genome (“+”) or knocked out (“−”); genomic DNA was cut (“+”) or uncut (“−”) with EcoRI prior to Southern blot analysis. The images shown in (**D**–**G**) represent four different blots and contain all telomeric bands (only irrelevant lanes were cropped out from the full-length blots). Original (no contrast adjustment) Southern blots from (**D**,**F** and **G**) are shown in Supplementary Fig. S9.

In *S*. *cerevisiae* and *K*. *lactis*, deletion of the RCT domain is sufficient to disrupt telomere maintenance^41^; therefore, we created mutant strains with C-terminal truncations of HpRap1s (Fig. 4A). Deletion of 148 amino acids from HpRap1A (spanning the whole RCT domain) was well tolerated, since the mutant strain (A^1–465^) showed no growth defects at a normal temperature (37 °C). However, it did not grow at a high temperature (47 °C), implicating HpRap1A in cell viability (Fig. 4B). Attempts to remove the entire RCT (we tried to delete 215 or 168 C-terminal amino acids) of HpRap1B were futile – no viable transformants with the correct gene replacement were obtained. Strains with smaller deletions (B^1–552^ and B^1–526^, lacking 24 and 50 amino acids, respectively) could be isolated and maintained. However, both mutant strains grew slower at 37 °C in liquid culture, in addition B^1–526^ strain formed smaller colonies on agar plates (Figs 4B and S4). Western blot analysis revealed a severe reduction in HpRap1B levels in the B^1–526^ (but not in the B^1–552^) strain (Fig. 4C).

Southern blot analysis of length of telomere restriction fragments (TRF) in the isolated strains revealed that RCT removal in HpRap1A does not perturb telomere maintenance (Fig. 4D). In contrast, we observed even in the B^1–552^ strain a modest telomere elongation. In the B^1–526^ strain, telomeres were overelongated and extremely heterogenous (Fig. 4E). In fact, the telomere signal in the B^1–526^ strain was almost completely smeared across the lane, and a substantial portion of it migrated faster than 0,7 kb (much faster than the shortest TRF band in the wild type strain, Fig. 4F). Such short telomeric DNA fragments were observed in other yeast strains with increased telomere recombination^41,53^. Indeed, we found that disruption of the telomerase RNA gene (HpTER) in the B^1–526^ strain neither changed telomere length nor induced senescence at any time point analyzed (Fig. 4F), whereas in the wild-type background, viability loss and telomere shortening were observed during the initial passages after HpTER inactivation^38,44,54^. In contrast, HpRAD52 knockout led to almost a wild-type telomere pattern (Fig. 4F). Moreover, shorter than 0,7 kb telomere-containing fragments were observed in the B^1–526^ strain (but not in the B^1–526^/∆*HpRAD52* strain), even if the genomic DNA was uncut with EcoRI prior to Southern analysis (Fig. 4F), suggesting that these fragments are extrachromosomal. Thus, the B^1–526^ mutation induces recombinational activities at telomeres independently of the presence of active telomerase.

Interestingly, there was a modest telomere elongation in the B^1–526^/∆*HpRAD52* double mutant compared with the wild-type strain (even after cultivation for 13–16 passages, each passage ~20–25 cell divisions). Therefore, the B^1–526^ mutation may slightly facilitate access of telomerase to telomeres. A similar telomere length increase was observed in the B^1–552^ strain (Fig. 4G). Combining these observations with the drastically reduced amount of HpRap1B in B^1–526^, we assume that HpRap1B RCT might play a role in telomerase regulation and is affected to the same extent by both RCT mutations. Recombination is activated specifically in the B^1–526^ strain due to the lower HpRap1B protein level (hence lower telomere accumulation).

### Rap1 paralogues are located at ribosomal protein gene promoters in *H*. *polymorpha*

Our analysis of HpRap1 mutants indicates their potential requirement for cell viability similarly to ScRap1 and in contrast to CaRap1. Since another marked difference between *S*. *cerevisiae* and *C*. *albicans* is the ability of ScRap1 to activate RPG transcription, we decided to test whether one of the HpRap1 proteins can recognize RPG promoters.

**Figure 5:**
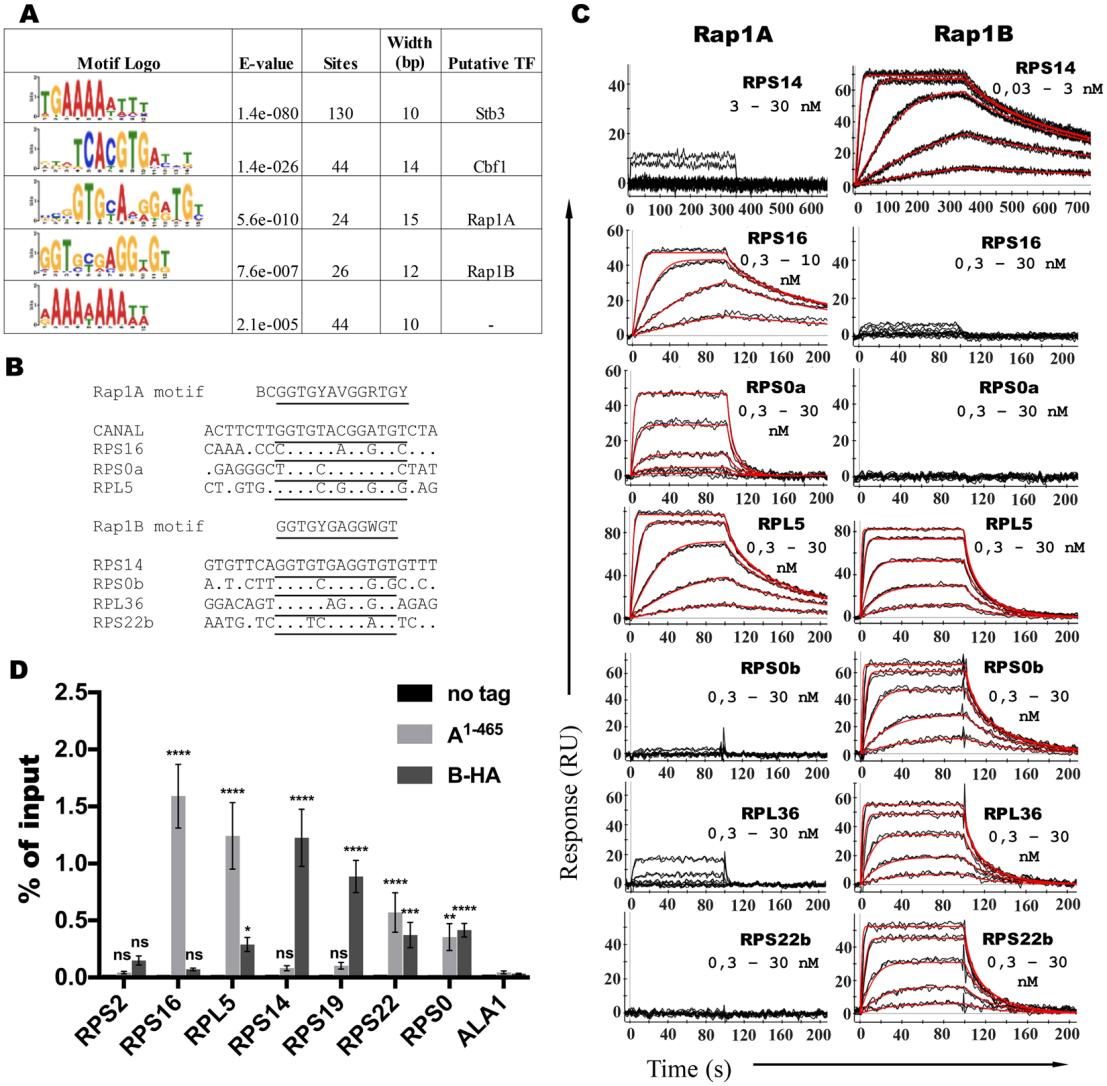
*H*. *polymorpha* ribosomal protein genes contain sites for loading of both Rap1 paralogues. (**A**) DNA motifs found to be enriched within HpRPG promoter regions using the MEME motif discovery tool. E-values, numbers of sites used for motif construction and width of the motifs are presented along with the putative transcription factors (TFs) associated with the motifs (see RESULTS). (**B**) Naturally occurring HpRPG promoter sequences tested in the SPR assay along with the discovered DNA motifs and high-affinity binding sites of either HpRap1A or HpRap1B (“CANAL” and “RPS14”) are presented. Lower case “a” or “b” in DNA names – sequence from the Rap1A or Rap1B motif, respectively (in cases in which a single promoter is predicted to contain both motifs). Core 12-13-bp sequences are underlined. (**C**) Sensograms showing the kinetic responses of HpRap1A (“Rap1A”) or HpRap1B (“Rap1B”) binding to the indicated DNA oligonucleotides (refer to (B) and Table 1 for sequences). The protein concentration range used in each case is indicated (0,03–30 nM means 0,03; 0,1; 0,3; 1; 3; 10 and 30 nM). Experimental data are shown in black, and fits into the Langmuir 1:1 kinetic model with mass transfer are shown in red. The obtained K_d_ values are shown in Table 1 and the full kinetic report in Supplementary Table S4. Three independent experiments were performed to obtain each K_d_. (**D**) ChIP analysis. Chromatin from the indicated strains was immunoprecipitated on anti-HA magnetic beads. DNA was analyzed by qPCR with primers targeting the indicated HpRPG gene locus as well as the control ALA1 locus. The amount of the DNA fragment in the IP samples is represented as the percentage of input DNA (mean ± SD); four replicates (cultures from four colonies of each strain) were used in the experiment. A two-way ANOVA test was used for statistical analysis of the difference between the means of the indicated test RPG and the control ALA1 gene. All the differences in the “no tag” control strain are nonsignificant. P-values (Dunnett’s multiple comparison test) (ns) <0,1234, (*) <0,033, (**) <0,002, (***) <0,0005, (****) <0,0001.

To this end, we used the *de novo* motif discovery algorithm (MEME) to identify potential *cis*-regulatory elements within sequences upstream of the *H*. *polymorpha* ribosomal protein ORFs. Five motifs that were significantly enriched within HpRPG promoter sequences were found (Fig. 5A, Supplementary Table S6). The most abundant TGAAAAWTTT sequence is highly similar to a ribosomal RNA processing element (RRPE), which was shown to be recognized by ScStb3 factor^55^. Similar motifs (GAAATTTT and GAAAATTTT, referred to as rapid growth elements (RGEs)) were found to be enriched in RPG promoters both in *S*. *cerevisiae* and *C*. *albicans*^37^. The core sequence of the next motif KKDTCACGTGAYYT coincides with the Cbf1 motif (tCACGTGca), which is enriched at CaRPG promoters (but not in *S*. *cerevisiae*); CaCbf1 is also involved in the regulation of CaRPGs^37,39^. Another discovered motif – RAAAAAAAWW – is similar to the *S*. *cerevisiae* poly(dA:dT) element, which is often present in yeast promoters (including ScRPGs) and influences transcription primarily via effects on nucleosome assembly^56^.

Most remarkably, the core 13-bp sequences within the VIIrAbs and *C*. *albicans* telomere (high-affinity sites for HpRap1A) are perfect matches to the first of the two remaining motifs – BCGGTGYAVGGRTGY, whereas the core 12-bp sequence of the *P*. *kudriavzevii* putative telomeric repeat (HpRap1B high-affinity site) differs from the second motif GGTGYGAGGWGT by only a single nucleotide. We measured the affinity of HpRap1B to a sequence from the promoter of the HpRPS14 protein (containing a perfect match to GGTGYGAGGWGT) by SPR and found that it was bound even tighter than the PICKU oligonucleotide (${{\rm{K}}}_{{\rm{d}}}^{{\rm{app}}}$ = 0.048 ± 0.007 nM for RPS14 vs ${{\rm{K}}}_{{\rm{d}}}^{{\rm{app}}}$ = 0.27 ± 0.08 nM for PICKU, Fig. 5B,C, Table 1). The identified HpRap1 RPG motifs are quite degenerate, and we decided to test several more RPG sequences for each motif (differing by 3 or more nucleotides in the core 12-13-bp sequences from the high-affinity binding sites (Fig. 5B)). All the tested oligonucleotides were bound by HpRap1A or HpRap1B, with apparent dissociation constants ranging from 0.5 to 10 nM (Fig. 5C, Table 1). Again, we observed less specific binding by HpRap1B, which bound sequence from the HpRPL5 promoter (predicted to be a HpRap1A site) with the same ${{\rm{K}}}_{{\rm{d}}}^{{\rm{app}}}$ as HpRap1A (Fig. 5C, Table 1). As judged by Western blot analysis, HpRap1 paralogues demonstrate a very similar cell abundance (Fig. 4C); therefore, at some sequences from the BCGGTGYAVGGRTGY motif, HpRap1s may actually compete for the same site.

Finally, we tested the above predictions *in vivo* using ChIP analysis. In total, 39 (53%) RPG promoters were predicted to contain either the BCGGTGYAVGGRTGY or the GGTGYGAGGWGT motif, and only 4 RPG promoters contained both sites (Supplementary Table S6). The promoter of the HpRPS2 (HPODL_05028) gene was predicted to lack both motifs, HpRPS14 (HPODL_01191) and HpRPS19 (HPODL_04662) – to contain HpRap1B sites only, and HpRPS16 (HPODL_04291) and HpRPL5 (HPODL_00846) – HpRap1A sites only, whereas HpRPS0 (HPODL_04762) and HpRPS22 (HPODL_01192) were expected to be bound by both proteins (Supplementary Table S6). The ChIP results confirmed these predictions, except for the HpRPL5 gene promoter – which was found to be occupied by both HpRap1 homologues (Fig. 5D). This result might provide evidence of competition for the same site, since (as discussed above) HpRap1 paralogues exhibited very similar affinity *in vitro* for the HpRPL5 oligonucleotide (Table 1). Thus, a significant portion of ribosomal protein genes might be regulated by Rap1 homologues in *H*. *polymorpha*.

## Discussion

Despite the great diversity of telomeric sequences in yeasts, chromosomal ends in the members of the two large subgroups of *Saccharomycotina* subphylum are recognized, bound and protected by the homologues of Rap1 protein^57^. Our results suggest that the methylotrophic yeast *H*. *polymorpha* DL-1 is no exception, although this species contains two Rap1 homologues (HpRap1A and HpRap1B) with considerably divergent primary structures. Remarkably, only HpRap1B protein is able to distinguish the cognate telomeric repeat *in vitro*, as revealed by our DNA binding assays. Moreover, we show herein that it also localizes near chromosomal ends *in vivo* and is necessary for telomere length regulation, implying that Rap1B is a major telomere protein in *H*. *polymorpha* DL-1. Several other methylotrophic yeasts also have a second copy of Rap1, and the sequence analysis presented in this study (Figs 1C and S1) suggests that the two Rap1s are paralogues (i.e., evolved independently). Therefore, we speculate that the telomere-dedicated role of the Rap1B subfamily may be conserved throughout the “methylotrophs” clade. As the first evidence supporting this notion, our results show that *in vitro* putative telomeric repeats from several methylotrophic yeasts can be bound by HpRap1B, but not HpRap1A.

Quite unexpectedly, we find that *H*. *polymorpha* telomeric oligonucleotide is far from being the most optimal substrate for HpRap1B – for example, a sequence from the HpRPS14 promoter is bound ~200 times more efficiently (Table 1). This result suggests that the telomere protective function does not necessarily require very tight binding by a telomere protein. The same conclusion was obtained by another group studying the properties of a major component of *Y*. *lipolytica* telomere – Tay1 protein^58^. YlTay1 prefers the canonical TTAGGG sequence (which is presumably bound by Tay1 homologues in non-yeast fungi)^59,60^ to its cognate telomeric repeat^58^. These two examples appear to be exceptions (telomeric repeats are usually preferred targets for the cognate telomere factors), but they are very important for understanding the evolution of the diversity of telomeric sequences (particularly in budding yeasts).

In almost all budding yeasts studied to date, Rap1 is involved in the maintenance of normal telomere length homeostasis. However, whereas in two species from the *Saccharomycetaceae* subgroup (*S*. *cerevisiae* and *K*. *lactis*) the major consequence of Rap1 dysfunction is telomerase-dependent telomere elongation^44,61,62^, the long telomeres in the *C*. *albicans* (“CUG clade”) RAP1 null mutant appear to be specifically maintained by recombination^38^. We find that the deletion of 50 a.a. from the C-terminus leads to long and very heterogenous telomeres. Elongation of telomeres in this B^1–526^ mutant strain relies on recombination rather than telomerase – a phenotype very similar to *C*. *albicans ∆rap1*. A comparison of telomere length in the B^1–526^/∆*HpRAD52* and B^1–552^ strains (25 a.a. RCT deletion) does point to the possible involvement of HpRap1B in telomerase inhibition. However, even if such a mechanism operates in *H*. *polymorpha* cells, its contribution to length maintenance seems to be minor. Thus, we propose that outside the *Saccharomycetaceae* group of budding yeasts, Rap1 homologues restrict telomere length primarily via the suppression of telomere HDR.

The phenotypic difference between the two strains can be explained by the different abundances of HpRap1B protein: the B^1–526^ strain has a very small amount of HpRap1B, approximately 1/20^th^ of the B^1–552^ and WT levels. Therefore, the RCT domain is important for the normal accumulation of HpRap1B, affecting either the stability or expression of the protein. This phenomenon may explain why we could not isolate viable strains with longer C-terminal truncations (215 or 168 a.a.) of HpRap1B.

We were unable to detect a telomere phenotype in the strain with HpRap1A lacking the RCT domain, which is consistent with its inability to specifically bind *H*. *polymorpha* telomeric DNA. However, we identified high-affinity HpRap1A binding sites within subtelomeres in close proximity to the first telomeric repeat, suggesting potential functional significance at chromosomal ends. *S*. *cerevisiae* subtelomeres are also decorated with a multitude of different transcription factors^63^. One such example – ScTbf1 protein – is particularly intriguing: it binds the canonical telomeric sequence (TTAGGG), and this binding influences telomerase recruitment and telomere capping^64–66^. However, this ScTbf1 role manifests itself at natural chromosome ends only when telomeres are short. Thus, HpRap1A telomere function may be revealed only at shorter telomeres.

In addition to its importance at telomeres, ScRap1 has been explicitly shown to be a central regulator of transcription^14^. Among the first identified and most prominent targets is the set of ribosomal protein gene promoters. Remarkably, the ability to regulate RPG transcription by Rap1 appears not to be conserved during evolution even within the *Saccharomycotina* subphylum^45^. *C*. *albicans* and *Y*. *lipolytica* RPG promoters are largely devoid of Rap1 binding sites, and motifs for Tbf1 and Cbf1 factors are instead predicted to be enriched within these sequences^37^. At least in *C*. *albicans*, Tbf1 and Cbf1 binding to the predicted sites has been confirmed *in vivo*^37,39^. Such Tbf1(Cbf1) to the Rap1 substitution at RPGs in budding yeast lineages is a classic example of a general process known as transcriptional rewiring, which mechanistically is still not very well understood^67,68^. Based on previous data, RP regulon rewiring has been predicted to occur in *S*. *cerevisiae* and its close relatives^37,69–71^; however, these studies did not include a single species from the “methylotrophs” clade. In the present study, we show that ~50% of RPG promoters in *H*. *polymorpha* are predicted to contain binding sites for one of the Rap1 paralogues. Here we demonstrate that the discovered sequences are capable of recruiting HpRap1 proteins *in vitro* and, most importantly, *in vivo*. Consistent with our results, putative sites for Rap1 were bioinformatically predicted to be enriched within RPG promoters in *H*. *polymorpha* and several other methylotrophic yeasts in a recent study by Sorrells *et al*.^72^. Therefore, RPG regulation by Rap1 factor is not confined to the *Saccharomycetaceae* lineage. Interestingly, Tbf1 sites (TTAGGG) were not discovered within HpRPG promoters, and although this finding may represent an artifact of the *in silico* prediction, we did found an element common to both *S*. *cerevisiae* and *C*. *albicans* RPGs (rapid growth elements (RGEs)) and the putative Cbf1 motif (an element specific to CaRPGs). Again, these findings are consistent with a recent report by Sorrells *et al*.^72^. Regulation of RPG transcription by Rap1 paralogues and (potentially) by Cbf1 in methylotrophic yeasts may thus represent an interesting intermediate between *Saccharomycetaceae* RPGs (controlled by Rap1) and “CUG clade” RPGs (controlled by Cbf1 and Tbf1).

Collectively, the present results suggest that budding yeasts from the “methylotrophs” clade utilize a particular set of Rap1 functions (aside from RAP1 gene duplication) that are distinct from other *Saccharomycotina* subgroups. At telomeres, *H*. *polymorpha* employs Rap1B primarily to protect against telomere overelongation via recombination (similar to *C*. *albicans*), whereas in the *Saccharomycetaceae* subgroup (*S*. *cerevisiae*, *K*. *lactis*), telomerase is responsible for the longer telomeres in *rap1* mutants. *H*. *polymorpha* shares with *S*. *cerevisiae* the importance of Rap1 for cell viability and RPG regulation, in contrast to *C*. *albicans* wherein Rap1 is a nonessential protein and *Y*. *lypolitica* wherein the RAP1 gene is missing. Therefore, in contrast to the previous proposals, Rap1 recruitment to RPGs is not confined to the *S*. *cerevisiae* lineage. Since *Saccharomycetaceae* branched off prior to the separation of the “methylotrophs” and “CUG” clades (Fig. 1A), it is possible that the last common ancestor of these three subgroups had already recruited Rap1 at RPG promoters.

## Supplementary information

Supplementary methods and figures

Supplementary Tables

## Data Availability

The data associated with this report are provided in the figures and Supplemental Figures.
