# Supplementary material for: Functional duplication of Rap1 in methylotrophic yeasts
Source: Sci Rep. 2019 May 10;9:7196. doi: 10.1038/s41598-019-43595-8 (PMC6510891; doi:10.1038/s41598-019-43595-8)
Supplement: Supplementary file 1 — Supplementary methods and figures [file 41598_2019_43595_MOESM1_ESM.docx]

**Functional duplication of Rap1 in methylotrophic yeasts*.***

Alexander N. Malyavko^1^*, Olga A. Petrova^1^, Maria I. Zvereva^1^, Olga A. Dontsova^2,1,3^

^1^Faculty of Chemistry and Belozersky Institute of Physico-Chemical Biology, Lomonosov Moscow State University, Moscow, 119992, Russia.

^2^Center of Life Sciences, Skolkovo Institute of Science and Technology, Moscow, 143026, Russia

^3^Shemyakin-Ovchinnikov Institute of Bioorganic Chemistry of the Russian Academy of Sciences, Moscow, 117997, Russia

**Supplementary methods**

**Construction of templates for PCR amplification of integration cassettes**

For the construction of cassettes for C-terminal truncations (and concomitant HA-tagging), we generated pFA6a-3HA-HpURA3 plasmid by replacing the PmeI-BglII fragment of pFA6a-3HA-kanMX6 vector^1^ with the fragment carrying the *H. polymorpha* CBS4732 URA3 gene (PCR product #1). PCR products #2 and 3 were cloned at SalI/XmaI and PmeI/ClaI sites (respectively) of the pFA6a-3HA-HpURA3 vector for construction of the A^1-465^ strain. PCR products #4 and 5 were cloned at SalI/XmaI and PmeI/ClaI sites (respectively) of the pFA6a-3HA-HpURA3 vector for construction of the B-HA strain (vector pFA6a-Rap1B-3HA-HpURA3). PCR products #6 and 7 were cloned at BsiWI/SmaI sites of the pFA6a-Rap1B-3HA-HpURA3 vector for construction of the B^1-552^ (pFA6a-Rap1B^1-552^-3HA-HpURA3 vector) and B^1-526^ (pFA6a-Rap1B^1-526^-3HA-HpURA3 vector) strains. PCR product #8 (with the LEU2 marker gene, generated from the pCHLX vector^2^) was cloned at BglII/PmeI sites of the pFA6a-Rap1B^1-526^-3HA-HpURA3 vector (substituting HpURA3 for HpLEU2) for construction of the B^1-526^(LEU) strain.

HpTER knockouts (in B^1-552^ and B^1-526^ backgrounds) were generated as previously described^3^. For the HpRAD52 (W1QIW9) knockout (in the B^1-526^ background), a 2241-bp fragment of the *H.* *polymorpha* DL-1 genome containing the *HpRAD52* gene was PCR-amplified (PCR product #9) and cloned into pUC18 at the SmaI site. The XbaI/BglII fragment of the pUC18Rad52 vector was then replaced with the XbaI/BglII fragment of the pCCUR1 plasmid (with the *HpURA3* gene).

Cassettes for RAP1A and RAP1B disruption in diploid *H. polymorpha* CBS4732 were constructed by cloning PCR products #10 and 11, respectively, into pUC19 at the SmaI site. The PCR product #8 (LEU2 marker gene) was then cloned at the MscI site of the pUC19RAP1A plasmid and at the EcoRV site of the pUC19RAP1B plasmid.

**Bioinformatic procedures**

The sequences of Rap1 homologues were identified by BLAST search in the NCBI database. The sequence accession numbers are *S. cerevisiae* (CAA96118), *S. castellii* (XP_003677388), *K. lactis* (XP_453918), *C. albicans* (KHC54070), *C. maltosa* (EMG49340), *D. hansenii* (XP_458241), *H. polymorpha* DL-1 Rap1A (XP_013932968) and Rap1B (XP_013934573), *C. arabinofermentans* Rap1A (ODV86102) and Rap1B (ODV86572), *P. membranifaciens* Rap1B (XP_019015510), *P. kudriavzevii* Rap1A (KGK36455) and Rap1B (KGK39353), *C. boidinii* Rap1A (OWB67710) and Rap1B (OUM53063), *P. pastoris* Rap1A (ANZ73741) and Rap1B (ANZ74953), and *P. tannophilus* Rap1A (ODV95885) and Rap1B (ODV94928). Three sequences were predicted from whole-genome sequence shotgun contigs by ORFfinder because the annotated protein sequences were incomplete. The WGS sequence accession numbers are *D. bruxellensis* Rap1A (AHMD01000471, REGION: 20105-22369) and Rap1B (AHMD01000277, REGION: 4900-6684), and *P. membranifaciens* Rap1A (AEHA01000048, REGION: 910-3720). Sequence analysis was conducted in MEGA7^4^. Sequences were aligned using Muscle and shaded with BoxShade; a portion of the alignment with Rap1 paralogues from “methylotrophs” was analyzed by the Neighbor-Joining method to infer phylogenetic relationships. The evolutionary distances were computed using the Poisson correction method and are presented in units of the number of amino acid substitutions per site. The percentage of identity/similarity between sequences of the *H. polymorpha* and *S. cerevisiae* Rap1 homologues (Fig. 1B) were calculated using the BLAST GlobalAlign tool.

Species abbreviations: SACCE – *Saccharomyces cerevisiae*, SACCA – *Saccharomyces castellii*, KLULA – *Kluyveromyces lactis*, CANAL – *Candida albicans*, CANMA – *Candida maltosa*, DEBHA – *Debaryomyces hansenii*, HANPO – *Hansenula polymorpha* DL-1, DEKBR – *Dekkera bruxellensis*, CANAR – *Candida arabinofermentans*, PICME – *Pichia membranifaciens*, PICKU – *Pichia kudriavzevii*, CANBO – *Candida boidinii*, PICPA – *Pichia pastoris*, PACTA – *Pachysolen tannophilus*.

*In silico* prediction of cis-regulatory elements within *H. polymorpha* DL-1 RPG promoters was performed using the MEME online tool^5^. To obtain the list of genes encoding HpRPGs, we first searched the UniProtKB database using the GO term “structural constituent of ribosome [3735]” (organism “*Ogataea parapolymorpha* DL-1”), which resulted in 127 genes. Genes annotated as components of mitochondrial ribosome were removed, and ambiguous annotations were manually reviewed (by BLAST search). The 73 remaining genes were utilized in this study (Supplementary Table S7). Genomic sequences upstream of start codons (-800 to -1) were extracted using the “RSAT retrieve sequence” online tool^6^. These sequences were used as input for MEME, which generated motifs enriched relative to a simulated 2-order background model. The complete results of the MEME search are shown in Supplementary Table S6.

**Protein Expression and Purification**

HpRap1A and HpRap1B were cloned into a pET30aTEV vector (kindly provided by Daniela Rhodes (MRS LMB, Cambridge, United Kingdom)) with TEV-cleavable N-terminal 6His- and S-tags. The resulting plasmids were transformed into *E. coli* BL21 (DE3) cells. Protein expression was induced with 1 mM IPTG at 18 °C overnight. The cells were collected by centrifugation, resuspended in lysis buffer (50 mM HEPES-NaOH, pH 7.5, 500 mM NaCl, 10 mM β-ME, 10% glycerol, 0.05% Tween 20, 30 mM imidazole, 1x Halt protease and phosphatase inhibitor cocktail, EDTA-free (Thermo Fisher Scientific)) and lysed by sonication. Protein fragments were purified using Ni-NTA-agarose (Sigma). The buffer was replaced with 50 mM HEPES-NaOH, pH 7.5, 100 mM NaCl, 1 mM DTT, 10% glycerol, 0.05% Tween 20, 5 mM imidazole, and 0.3 mM PMSF on PD Minitrap G-25 columns (Sigma), and the 6His-S-tag was excised with recombinant TEV protease (50 mkg per 1 mg of target protein), incubating 2 h at room temperature. Tag and TEV protease were removed by an additional round of affinity chromatography on Ni-NTA-agarose (Sigma): most of the HpRap1A protein was in the flow through fraction, whereas tag-free HpRap1B was bound to Ni-NTA under these conditions and was eluted from resin using buffer consisting of 50 mM HEPES-NaOH, pH 7.5, 500 mM NaCl, 1 mM DTT, 10% glycerol, 0.05% Tween 20, 50 mM imidazole, and 0.3 mM PMSF. The buffer was the replaced with SPR0 buffer (10 mM HEPES-NaOH, pH 7.5, 150 mM NaCl, 0.5 mM DTT, 0.05% Tween 20) on PD Minitrap G-25 columns (Sigma) and stored at +4 °C. Protein concentrations were determined spectrophotometrically by the absorbance at 280 nM (extinction coefficients were calculated using the ExPaSy ProtParam tool^7^).

**Random spore analysis**

The heterozygous strains RAP1A^+/-^ and RAP1B^+/-^ were sporulated on maltose-containing medium (2% maltose, 3% agar) for 10 days at room temperature. Sporulated cells were resuspended in 0,75 ml of sterile water (OD_600_ ~0.5) in 2-ml Eppendorf tubes, mixed with 0,75 ml diethyl ether and rotated for 30 min at room temperature. The organic phase was removed, and the cells were diluted (1/10 and 1/100) in water and plated on YPD. After 2 days of growth at 37 °C, 96 random colonies (48 from RAP1A^+/-^ and 48 from RAP1B^+/-^ strains) were analyzed for the presence of three genetic markers (LEU2, ADE2 and URA3): yeast cells were resuspended in water (OD_600_ ~0.01) and plated on the appropriate selective plates containing SD medium (0.17% yeast nitrogen base, 0.5% ammonium sulfate, 2% glucose, 2% agar) supplemented with leucine (100 mg/L), adenine hemisulfate (20 mg/L), and uracil (20 mg/L). An additional 20 random colonies from the YPD plate (10 from RAP1A^+/-^ and 10 from RAP1B^+/-^ strains) were inoculated in 1 ml of YPD, grown overnight at 37 °C and (after genomic DNA extraction), and subjected to PCR analysis using the Q5 polymerase. Primers for the RAP1A gene: Af (5’-TGCCATCATGGAGAAGAATTCG-3’) and Ar (5’-AGCCTGGTAGATGGAGTAGCC-3’); primers for the RAP1B gene: Bf (5’-GTCGACTCGTCGAGAATCGAG-3’) and Br (5’-TCTTCTCGTGGAAGAACTGCC-3’).

**Supplementary references**

1. Longtine, M. S. *et al.* Additional modules for versatile and economical PCR-based gene deletion and modification in Saccharomyces cerevisiae. *Yeast* **14**, 953–961 (1998).

2. Sohn, J. H. *et al.* A novel autonomously replicating sequence (ARS) for multiple integration in the yeast Hansenula polymorpha DL-1. *J. Bacteriol.* **178**, 4420–4428 (1996).

3. Smekalova, E. M. *et al.* Specific features of telomerase RNA from Hansenula polymorpha. *RNA* **19**, 1563–1574 (2013).

4. Kumar, S., Stecher, G. & Tamura, K. MEGA7: Molecular Evolutionary Genetics Analysis Version 7.0 for Bigger Datasets. *Molecular Biology and Evolution* **33**, 1870–1874 (2016).

5. Bailey, T. L. & Elkan, C. Fitting a mixture model by expectation maximization to discover motifs in biopolymers. *Proc Int Conf Intell Syst Mol Biol* **2**, 28–36 (1994).

6. Medina-Rivera, A. *et al.* RSAT 2015: Regulatory Sequence Analysis Tools. *Nucleic Acids Research* **43**, W50–W56 (2015).

7. Gasteiger, E. *et al.* Protein Identification and Analysis Tools on the ExPASy Server. in *The Proteomics Protocols Handbook* (ed. Walker, J. M.) 571–607 (Humana Press, 2005). doi:10.1385/1-59259-890-0:571

SACCE 1 --------------------------------MSSPDDFETAPAEYVDALDPSMVVVDSGS-------------------
SACCA 1 -------------------------------------------MSSPQNFETAQQYMDSLS-------------------
KLULA 1 --------------------------------------------------------------------------------
CANAL 1 --------------------------------------------------------------------------------
CANMA 1 --------------------------------------------------------------------------------
DEBHA 1 --------------------------------------------------------------------------------
HANPO_Rap1A 1 --------------------------------------------------------------------------------
DEKBR_Rap1A 1 --------------------MPKKSSSSAKRKAKTSSGQEEQDDHSLVSLTTLDNSQVAAA-------------------
CANAR_Rap1A 1 ------------------------------------------------------MSSDSLA-------------------
PICME_Rap1A 1 MFLVTETSQHHQNIQRDTSVDKDRALKFFEKYIGALYPLGTMAGRDKAHKQRDRASQNKAG-------------------
PICKU_Rap1A 1 -----------MVLKDQIPAKDTKVSQATQSGSATKEPLRKKSNHENVNVENSHIRSTIAK-------------------
CANBO_Rap1A 1 MSDTKQDTKKQEEVVKEVDQSSAVAPATAPEVEKTKEVTKTDADADATAAATAAAVAAIQASGIIPDEKPTAKSTTTSTT
PICPA_Rap1A 1 --------------------------------------------------MIKRPSNNSLT-------------------
PACTA-Rap1A 1 --------------------------------------------------------------------------------
HANPO_Rap1B 1 --------------------------------------------------------------------------------
DEKBR_Rap1B 1 --------------------------------------------------------------------------------
CANAR_Rap1B 1 --------------------------------------------------------------------------------
PICME_Rap1B 1 --------------------------------------------------------------------------------
PICKU_Rap1B 1 --------------------------------------------------------------------------------
CANBO_Rap1B 1 -------------------MSETERIMVLSTPAEKSSELDAQLDASSNDKKISDNLSNIGD-------------------
PICPA_Rap1B 1 --------------------------------------------------------------------------------
PACTA_Rap1B 1 --------------------------------------------------------------------------------


SACCE 30 ---------------------------------------------------------------------------AAVTA
SACCA 19 ---------------------------------------------------------------------------DSNNS
KLULA 1 ------------------------------------------------------------------------------MS
CANAL 1 --------------------------------------------------------------------------------
CANMA 1 -----------------------------------------------------------------------------MNY
DEBHA 1 ---------------------------------------------------------------------------MFHTI
HANPO_Rap1A 1 ---------------------------------------------------------------------------MASND
DEKBR_Rap1A 42 ---------------------------------------------------------------------------VLAEV
CANAR_Rap1A 8 ---------------------------------------------------------------------------IENNS
PICME_Rap1A 62 ---------------------------------------------------------------------------TYGNN
PICKU_Rap1A 51 ---------------------------------------------------------------------------QSKGK
CANBO_Rap1A 81 PSTTTTNQLLKNKSFYIISKDNSSETIKQLSNKIEKLGGLIKTNLNDEFKGTILAENKESIPSSISQKVLSFEYINELSN
PICPA_Rap1A 12 ---------------------------------------------------------------------------DLSVR
PACTA-Rap1A 1 --------------------------------------------------------------------------------
HANPO_Rap1B 1 --------------------------------------------------------------------------------
DEKBR_Rap1B 1 --------------------------------------------------------------------------------
CANAR_Rap1B 1 ---------------------------------------------------------------------------MDETA
PICME_Rap1B 1 --------------------------------------------------------------------------------
PICKU_Rap1B 1 --------------------------------------------------------------------------------
CANBO_Rap1B 43 ---------------------------------------------------------------------------KVNEN
PICPA_Rap1B 1 --------------------------------------------------------------------------------
PACTA_Rap1B 1 --------------------------------------------------------------------------------


SACCE 35 PSDSAAEVK------------------ANQNEENTGATAAETSEKVDQTEVEKKDDDDTTEVGVTTTTPSIADTAATANI
SACCA 24 HMENQQQQD------------------ASPNANGNGAVTTEETNVDPSITNVKMEGTNNADADITIALPATQDVNNDSNG
KLULA 3 HADDFDTAL------------------DSPSVQ-----------------------------------------------
CANAL 1 --------------------------------------------------------------------------------
CANMA 4 NYTTFDDDD------------------GRYTTDLSEATARAAEQAAGQQQQQYQLNRRTQMEAATRQAQELSKEVEKPKM
DEBHA 6 EYSAGMVSP------------------NSNNQLALGNVQSGAAASHG---------------------------------
HANPO_Rap1A 6 AESSLESAA------------------ATATAAMKASEQEKTSNAVLEDESLINNLVESLDKPDDTDASIKAEESL----
DEKBR_Rap1A 47 AKEASGTLG------------------DMMEDEKRDNSKRNHVNKTAQGKNKKGKHRATKDSPAPEKNEDASGVNREEKE
CANAR_Rap1A 13 SSTVLEPAI------------------DASNAAEAAAAAVAALEAKEQSEASKDSNGKASSSSTSSTTPPIDESVVDPDL
PICME_Rap1A 67 EGETAAALQ------------------ESNIETQFQNQIQSQLQNQLQNELSHTFENHEEADGHSTANVNILDSTNIDDE
PICKU_Rap1A 56 LADDDTNAE------------------DNNIETQFQNQIKSQLQNQLQSELHHSFHKDNESNPQNSTVDNLLDSTNIDDE
CANBO_Rap1A 161 SNNPIEKTSKFILKKSDDHHGSGSGAGGDDSSEKKKDKTKDSKEKKDKKSEKKDKKDKDKKEKKDKDKKEKKDKEKKEKL
PICPA_Rap1A 17 EILTFKAKT------------------NNKKKTTVTQTPQQTLYLLPPNTNHQEMSEPNTKKQKLTPYKN----------
PACTA-Rap1A 1 ---------------------------MTEITDLNT--------------------------------------------
HANPO_Rap1B 1 ----MDSML------------------HQQIAQYPGAGPAQSD-------------------------------------
DEKBR_Rap1B 1 ---------------------------MSSLTASNDDQ------------------------------------------
CANAR_Rap1B 6 ADQSIFTEI------------------TEGAAELIPENPKVDTNASEVAVYEDGLKD-----------------------
PICME_Rap1B 1 ---------------------------MSGKSD-----------------------------------------------
PICKU_Rap1B 1 ---------------------------MAPKTD-----------------------------------------------
CANBO_Rap1B 48 ETNNNDAKKY-----------------DNGIEDKSGRLDEQAIKEAELKEPAEAVDKETAEKQDVENEGALIADKEVEKE
PICPA_Rap1B 1 -MNNFEDIQ------------------VNQVTSPIAHNRKTEA-------------------------------------
PACTA_Rap1B 1 -MTDFVSLP------------------DSNKESPHKNSSANYDHE-----------------------------------

**BRCT**

SACCE 97 ASTSGASVTEPTTDDTA------------------------ADE-KKEQVSGPP--------------------------
SACCA 86 TAAADKDGNSPNNE-------------------------------TSTSTGENP--------------------------
KLULA 18 ---------------------------------------------IKEDVGVFD--------------------------
CANAL 1 --------------------------------------------------------------------------------
CANMA 66 RGRFDPPKT------------------------------------IFVDKDNKT--------------------------
DEBHA 35 ---------------------------------------------IFTYDGGIP--------------------------
HANPO_Rap1A 64 ---------------------------------------------VDPSLSRSP--------------------------
DEKBR_Rap1A 109 IKQETKEEVSNTEKDEN------------------------SEEEKRPGNEKKP--------------------------
CANAR_Rap1A 75 PKDE-----------------------------------------ITTKSNGDD--------------------------
PICME_Rap1A 129 LRNLEQLDINNIAKSAV---AAAAVSFAQ-AATADDINHAIEGGDAENDVEGAEEEEVAAKTEINEGDDRGMTDEDEVIK
PICKU_Rap1A 118 LRNLENMGIDNLSETVVQEAAAAVVSFAQAAATANNMNESGNIDVELAEIDTNI--------------------------
CANBO_Rap1A 241 DKKSDKKDKSDKIEKLDKKDKSDKKDKSDKIEKLDKKTDKSNKSSTVDDSTTTP-------------------------K
PICPA_Rap1A 69 ---------------------------------------------ILIDNVGNP--------------------------
PACTA-Rap1A 10 ---------------------------------------------LFTTDDNRP--------------------------
HANPO_Rap1B 22 ---------------------------------------------VFIDATGKP--------------------------
DEKBR_Rap1B 12 ---------------------------------------------LFVGPDGTS--------------------------
CANAR_Rap1B 45 ---------------------------------------------LFTDLNSKP--------------------------
PICME_Rap1B 7 ---------------------------------------------IFVDAAMKP--------------------------
PICKU_Rap1B 7 ---------------------------------------------IFVDAGLKP--------------------------
CANBO_Rap1B 111 ADYESAKPISRSRPDIA--------------ELLLNLRDYIPASKLFTDIDSNP--------------------------
PICPA_Rap1B 25 ---------------------------------------------IFVTPIGTP--------------------------
PACTA_Rap1B 27 ---------------------------------------------LFTNALNEP--------------------------

**BRCT**

SACCE 126 ----------------------------------LSNMKFYLNRDADAHDSLNDIDQ----LARLIRANGGEVLDSKPRE
SACCA 109 --------------------QKDQNATPVAISTLFQGMNFFMNRNNDAHDSAHDVDQ----LARLIMAHSGNVLASLPED
KLULA 27 ------------------------------------GVSFFID------PLINDMEA----LGNAVRNNGGAVLIEAPEK
CANAL 1 -------------------------------------MLFYIP------EDEPNRNK----YRDLIIRYGGVIVEKGF--
CANMA 84 -------------------------------------MLFYIP------KEEPNRIK----YRELIIKHGGLLVDKQI--
DEBHA 44 -------------------------------------MLFFVP------FNDPNRYE----YKQKIISHGGIITDTEPNE
HANPO_Rap1A 73 ------------------------------LDQVLLGQKFILL------GNGAANED----VSHVIEELGGSVVSGEN--
DEKBR_Rap1A 139 ----------EVAKDNSNEQELITRPNNANFSHLLEGKTFAIY------GKTDAVKD----VKMMVESLGAKITNEMG--
CANAR_Rap1A 88 --------------------ENKLTDPSTTISELLKGTKYYII------SNTFAKKD----ITKLIKVLGGEVSDVLS--
PICME_Rap1A 205 EVVKEQGVELSADGNQDDDDADMKTPSSTDFSLLLKGLVFSLP------VFSEENDH----LKTMITSLGGEVVDDGN--
PICKU_Rap1A 172 ---------NSNHDNISVSDNVDQKLTAHNQERIFKGKVFYIP------YDSPDNTN----IKSMILQMGGKLVDSST--
CANBO_Rap1A 296 TTEKSDEKTVPSEDKKEKKEKKEKKDDDKNGDLKPSSESTSTT------ATADADTE----AAAAKEAAKAAATAAATAA
PICPA_Rap1A 78 -------------------------------------LSFHIL----GYFTKSDRSQ----LINMILEMGGKVVPDLPSP
PACTA-Rap1A 19 -------------------------------------YVFIVS------ETIQNRSE----VVSLINEYGGRVLDNGSHN
HANPO_Rap1B 31 -------------------------------------MKFYLY------PPLDRNQQ----LINLIENNGGHVVAGVS--
DEKBR_Rap1B 21 -------------------------------------YIFYLC------PPLNTNDL----LIKMIEEHGGGVTYDLE--
CANAR_Rap1B 54 -------------------------------------MKFHLC------LPVPEH------ILEYITKGGGEVVNEYNG-
PICME_Rap1B 16 -------------------------------------YNFYLC------PPLNRSER----LVDMVEDNGGRVVHDFL--
PICKU_Rap1B 16 -------------------------------------YNFFLC------PPLDRSER----LIRMIKDNGGEVVQDFL--
CANBO_Rap1B 151 -------------------------------------LIFYLA------PPFDKNKE----MITLITINGGEIANSVESP
PICPA_Rap1B 34 -------------------------------------YKFFLV------GKEDSGKEVEAVYKERITQHGGTLIDSTDD-
PACTA_Rap1B 36 -------------------------------------YVFLIP------EDEPELIK----LTKLIQEEGGRVINRLLPE

**BRCT**

SACCE 168 S-------KENVFIVSPYNHT---------NLPTVTPTYIKACCQSNSLLNMENY-L----VPYDNFREVVDSRLQEESH
SACCA 165 TTD-----VSNVYVISPYNDT---------KLPTVTPTYIKACVSNNTLLNINHY-L----VPYDEFRSVIDTQLQSETN
KLULA 61 SSREW---EAAYFVSKRYDED---------YRIFVHPSYILDCIDAGTLLNVHDY------LGKPESSGFVRFDSNSISD
CANAL 32 --------PTVILLSNNED-----------LYGFSKLKFIDDCIAKNQLLSVFDYGTYG--TYNNPEDAAFDTTSIINEL
CANMA 115 --------PTAYHLSNNEK-----------MLGYFKLKFIDDCIANGGLVPLLDYGT----YNIPEDAGAFDP-----LI
DEBHA 77 ST------GNRIILLSSFNIR---------GQLCFKLSFIDDSIKNNGVCDLSNY-K----YQIPEVY------------
HANPO_Rap1A 111 --------NQGIYLARSVSDVNPNI-----NVPVYSFNFVYTAQRDHVLPDLNDF-K----IRRPP------------QL
DEKBR_Rap1A 197 --------PGVIPLVRSASTAVPAE-----FDDGYTFDLIYSIHKNGKIPSLEEF-K----VKRPP------------NL
CANAR_Rap1A 136 --------DDSYLLVRSASDIPLDK-----EVDAYSFNYIYAVHSNKILPDIEEY-K----LRKPP------------QL
PICME_Rap1A 273 --------LDALALVPSKEKSRTD------LAYQYLYQYIHDTFSKKTPLDINKY-R----VGK--------------SS
PICKU_Rap1A 231 --------PSSITLAPPNFKGPLN------ISNQFSYQFIYDSYIHKSLKDITKY-K----VNKSDLIEATAVEPTIEAK
CANBO_Rap1A 366 AAAA----VAAVTSIAPSHPEPSSSA----SIAESQDSKISESTTDATAPSTSTT-TTTTATKKESKKEAKKEKDAIVKK
PICPA_Rap1A 113 V-------DGVLFLTGDYKSIDEDTLKQLGDVPIYRDSFIYQCFVHKTSLPIDTF-----------------------RI
PACTA-Rap1A 52 G-------KGIIFLTARNSDEDILPQ----GKIIYDVNFVYDSIRSKSKLDLNDY-------------------------
HANPO_Rap1B 62 --------KDTVILSSEEFLMSEDLK----RFHVYRYEFVQKSITHG-IQNLSDY-E----IHNP---------------
DEKBR_Rap1B 52 --------HTCIVISVPGNVPDELR-----SAHIYSYKVIEDSVREGIQQEFGRY-L----IHNPEVEDIVQG-----EV
CANAR_Rap1B 84 --------PDIILIADPRGNFRTVYE----HLKIVSYLFVEDSYKKGTLMRLVDY-T----VHDPN--------------
PICME_Rap1B 47 --------QNCIVISSPDYVVPEDLK----TAHIYSYEVIQDSANRGAQQEFANY-L----IHVPTKNRESVPHQYSERK
PICKU_Rap1B 47 --------QNCIIISSRDYKIPEELK----SAHIYSYEVIQDSANRGAQQEFSNY-L----IHIPDANNDTMPHQFHDDN
CANBO_Rap1B 184 VESRVAEGPNAPIPIASPASIPQDMK----QVRFYSYKFILESIGKGMQLDLTNYLIAGTGIPEDVIDAAAVAEAALNAD
PICPA_Rap1B 70 --------PLTLFLAESQEILNRNLQTVPNNRQVYRLSFIDDCINEGIVLPLENY-T-----------------------
PACTA_Rap1B 69 DIKENPQILTSIIVLTSSEDYDDPFIP---SSRYFNPIYVFECIRERTTLNLNYF-K----LVNPTKEPKVVSVSPSKEA
SACCE 227 SNGVDNSNSNSDNKDSIRPKTEIISTNTNGATEDSTSEKVMVDAEQQARLQEQAQLLRQHVSSTASITSGGHNDLVQIEQ
SACCA 226 NDDDHVDHDEASKKRNLFEADPEEATQLNSTEPQGTSTQLPQDN-----------------KTASNPTTISAQPENNQEA
KLULA 123 EYNIGSQLS-----------------DLPGSDVTRAAVKIATGI-----------------NESIDSENTRGQREMDSDS
CANAL 91 NDDLSSNISS----------------SFSTIEGTSSMVEVPVAA-----------------PAAIPLPPPPHQQQRSIDA
CANMA 167 MNDLSSDITLE---------------QFPMENASTGKFTPILSS------------------------------------
DEBHA 125 --------------------------DPSSVSGQNKVIKINSEG-----------------DGADQNDAAAFSNVLSRN-
HANPO_Rap1A 161 PQNLNINIG-----------------ELAKTGLKEDESVESALS-----------------MSAEP--------------
DEKBR_Rap1A 247 PGNVKINTSDL---------------TDAEKKDREADIKSVAES-----------------LPLPPAPHPAAKRTRKASA
CANAR_Rap1A 186 PSHLKINVG-----------------DLASSEPDSDKSLSISDE-----------------ISSKPPTKVSKTRNSKTNT
PICME_Rap1A 320 GQSLNINIG-----------------EFPIKPVQDEKLNENGDG-----------------IDANPFDFVSGDSNALDSK
PICKU_Rap1A 292 DTSPLLAPTAPASS------------ASPATPVKESGSKLLKIN-----------------VGEFPLPKESAIESETSFD
CANBO_Rap1A 437 EKEIKKEK------------------EAAIKKDKSTKESTVARI-----------------SRIDPSKPFNEDDYKNINL
PICPA_Rap1A 163 DKNVDLAQ------------------DLINRALQESVDHVTSAS-----------------TAAAAAVVVATNGLS----
PACTA-Rap1A 96 --------------------------KIPTSGGDDNTAK-----------------------------------------
HANPO_Rap1B 109 --------------------------AEPSAPSPLTNISSPATM------------------------------------
DEKBR_Rap1B 109 DENGNTVIGAPDEG------------DMRSGSHLEDDNGFNTET-----------------NDLQVLPSTISASLAT---
CANAR_Rap1B 133 --------------------------------------------------------------------------------
PICME_Rap1B 110 DQPGAGGGD-----------------DLSGIGGTGDDYNFDVPG-----------------HGHAGAGEVSGNGNGNGNG
PICKU_Rap1B 110 AENVEAGIVVDGDGNYDFENLSKS--LVPANLSHAEEVNEIDVI-----------------KDLENNSHLSIDFSEHVEQ
CANBO_Rap1B 260 SSKPDNNVDSG---------------ETGDANGDTGALVHIPDA-----------------RDIAIIPE-----------
PICPA_Rap1B 118 --------------------------LLVDTNAQVYKMTPV---------------------------------------
PACTA_Rap1B 141 GSKHEITTPVKSK-------------EAPNAKARSAAAATATAT-----------------AATAAAVSNSASDSAAGTI

**Myb1**

SACCE 307 PQKDTSNNNNSNVNDEDNDLLTQDNNPQTADEGNASFQAQRSMISRGALPSHNKASFTDEEDEFILDVVRKNPTRR-TTH
SACCA 289 NNAAQQQTEEQLRLLQEHAQEVNQQAEPSNVSDEDVNYEERAYMMRAALPSHNKASFTEAEDEFILDVVRKNPTRR-TTH
KLULA 169 SPEVKMSSKDDEIASHDQQQQLTHTDVDDANDLILH---------EQASSSHNKSSFTKEEDEFILDVVRKNPTKR-TTH
CANAL 138 FPNPNI---------------------------------------NNARRGSSGHRFTIEQDEFILEHIRRKPRFR-QSQ
CANMA 196 ---------------------------------------------AGSSKRKGTNRFTAEQDEFILEQVRMKPRYR-HSH
DEBHA 161 ---------------------------------------------VRPPPMRTSRRFTDLKDEYILKQIRLNPRLR-NSH
HANPO_Rap1A 193 -------------------------------------------------QKKRSMKFTELEDACILDLIRRNPNLR-STH
DEKBR_Rap1A 295 KSKSGLANADS----------------------------------EYTTPKKRSSKFTVTEDEAILDLVRRNPYLR-STH
CANAR_Rap1A 232 TNAHPLLPPAEY---------------------------------LAATPKKRSSKFTPQEDEAILDLIRRNPNLR-STH
PICME_Rap1A 366 PSSKKKVTKK-----------------------------------HDPTKAKKSNKFTKEEDDFILDLVRRNPHLR-STH
PICKU_Rap1A 343 EPTTDLKNKRRIIKN------------------------------PNPQPKRKSKKFTPEEDEFILDLVRRNPHLR-STH
CANBO_Rap1A 482 YPGALLPPS------------------------------------KISPQNKKSIKFTSVEDEVILELIRRNPHLR-ATH
PICPA_Rap1A 204 ---------------------------------------------SKPDARTSKIQFTPEEDRFILDFVRRNPKRR-NTH
PACTA-Rap1A 109 -------------------------------------------------KKAQKNNFKPEEDEMILELIRQNPTLR-DTH
HANPO_Rap1B 127 ------------------------------------------------------RYFTQEEDNVLFEEIRKRPWMGYKGH
DEKBR_Rap1B 157 ---------------------------------------------AVFPRRHGIKYFTPEEDAFLLEEIRKRPWLGFRGH
CANAR_Rap1B 133 -------------------------------------------------ARSALRYFTPEEDAYLFEEIRKRPWFGYKGH
PICME_Rap1B 156 NGADINDGDDGVEAEEAGSNHLGVHGSELIFPHNKSEGS------IANYDDHSVRYFTDEEDKVLMEEIRKRHWMGIKGH
PICKU_Rap1B 171 YENTEVGGTERVGMNEEEQQPMSNVKFH-----------------PSVYDDYSVRYFTDDEDKVLMEEIRKRHWMGIKGH
CANBO_Rap1B 297 ---------------------------------------------NGKQGKAPVRFFSAQEDHMLLEEIRKRPWMGFRGH
PICPA_Rap1B 133 -----------------------------------------------------RSYFTKEEDNIIAETYRLLIPTH-RPV
PACTA_Rap1B 191 GEGTPISSSQAGR--------------------------------QAQENLARKNAFTEEEDEYILEEVRKHPRNR-KGY

**Myb1**

SACCE 386 TLYDEI--SHYV--PNHTGNSIRHRFRVYLSKRLEYVYEVDKFGK-LVRD-DDGNLIKTKV-------------------
SACCA 368 TLYDEI--SHYV--PNHTGNSIRHRFRVYLSKRLDYVYQVDSYGK-LVRD-ENGNLIKTKT-------------------
KLULA 239 TLYDEI--SHYV--PNHTGNSIRHRFRVYLSKRLEFVYQVDEDGK-LVRD-QDGNLIKTDI-------------------
CANAL 178 KFYAQLALLEPL--RGHTGNSIRSRFRKHLESRLNYIYKTDDQDQ-LIRD-EAGNLIKIGLDE-----------------
CANMA 230 QFYHDLSQTREL--HGHTGNSIRSRYRRHLEPLLSFVYKTDDRDR-LMRD-GNANLIKVGLNE-----------------
DEBHA 195 KFFDGLALHDVL--KGHTGNSIRSRYRNHLESRLAYVYKTDEDGV-LIKS-ADGKNIRDSLEN-----------------
HANPO_Rap1A 223 SFFAKIAQLPVL--SGHTGNSIRFRFRKILADKLDYVYQVDPQTNKLILDPVTNEPIKVKD-------------------
DEKBR_Rap1A 340 SFYAQIAQIPLL--SNHTGNSIRFRFRKILSKRIEWVYKVDPATNEIQLDPETNEPIKVKH-------------------
CANAR_Rap1A 278 SFYARIAQLPLL--SSHTGNSIRFRFRKILQPRLEYVYQLDPETNEVMINSETGKPIIIKD-------------------
PICME_Rap1A 410 TFFARIAQLKPL--SEHTGNSIRYRYRKVLAPNLAFVYKIDPKTGKPEIDAATNQPKKIED-------------------
PICKU_Rap1A 392 TFFARISQLAPL--SDHTGNSIRYRYRKVLAPQLDYVYEIDPVSGKPKIDPKTNAPIEVTE-------------------
CANBO_Rap1A 525 SFFTQIATLPSL--SGHTGNSVRFRFRKVITPNLDFVYDVDPKTNELKLDPETKEPIKIQE-------------------
PICPA_Rap1A 238 QLYTEL--AQHM--KNHTNHSIRHRFRRNLSAQLDWVYDIDPLTNQPRKD-ENGNYIKMQD-------------------
PACTA-Rap1A 139 FLFDKI-IQKSL--PNHTGNSIRYRYRTKLKDKLEYVYQVDEDGK-LVKD-VNDQYIKQTV-------------------
HANPO_Rap1B 153 QIYKDIAELDFFKERNRSAASLRERIRT-LKYDIEYVYKAGPNKE-LLRD-ANGNYIRDY--------------------
DEKBR_Rap1B 192 QIYKEIAAMDFFRKRGRSYSSLRERIRT-LKYHVGYVYKATKDHK-LLVD-ENGNYVRTY--------------------
CANAR_Rap1B 164 QIYREIAQLDFFVARHRTAASLRERIRT-LKYDIKYVYLESSNSRTLQKD-SQGRLIRDY--------------------
PICME_Rap1B 230 SIYEAISKMPYFVSRRRTPASLRERMRT-LKYKVGYVYKVDKKNR-LLKD-ASGSYIKTT--------------------
PICKU_Rap1B 234 LIYEAISELPYFKNRRRTSASLRERMRT-LKFNLGYVYQVDKNHN-LLRD-EKGNYIKTT--------------------
CANBO_Rap1B 332 QIYKDISETDYFKLTRRTPASLRERIRT-LRYDIQYVYKADSRNN-LLKD-KDGNYIKVY--------------------
PICPA_Rap1B 159 EINTHI-HKKYL--PHRTVYSIKSRYNEKIRKSLKYYYQSDPDSKELLRD-VEGNYVRVYINDADASTADDKASEEPLTS
PACTA_Rap1B 238 TLYNDI--SKNL--KNRTAVSIRSRFKQYLEPRLKYVYQVDSNNN-LIKD-SNNELIKTSDLA-----------------

**Myb2**

SACCE 441 --LPPSIKRKFSADEDYTLAIAV----------------KKQFYRDLFQIDPDTGRS-----------------------
SACCA 423 --LPPSIKKKFTADEDYILALAV----------------KKQFYRDLYQIDPDTGTN-----------------------
KLULA 294 --LPNGLKRKFTSEEDYNLAVAV----------------KKQFYRDAFQRDPDTGAS-----------------------
CANAL 237 --MPGTLKNKFTPEDDYLLCCVA-----------------LEYM-------ASNNID-----------------------
CANMA 289 --IPGTLKNKFTAEDDYFLC------------------------------SEAIKSE-----------------------
DEBHA 254 --LPKTLKNRFTALEDFNLCTEL------------IEYSRLKYYEDLESGAVDKDED-----------------------
HANPO_Rap1A 282 --LPSLLKSQYTAEEDYELCKHI-----------------LHFKENMSQYAKKRKLD-----------------------
DEKBR_Rap1A 399 --LPGLLKSQYTAEEDYNLCKAV-----------------QDFKENGSSLSTRKRRS-----------------------
CANAR_Rap1A 337 --LPELLKSQYTAEEDYELCKHI------------------RYFKDHSEDFLKKRKG-----------------------
PICME_Rap1A 469 --IPSLIKSQYTSEEDYALCKHI-----------------LLYKNGEMVLSGKKKHE-----------------------
PICKU_Rap1A 451 --IPSLIKSQYTSEEDYQLCKHI-----------------LAYKKGDLVLYGKKKHE-----------------------
CANBO_Rap1A 584 --LPELIKSQYTAEEDYILCSKILNFKQNASGITPASSSKLEPKESDESIEEDTEKDEKIGKEEPKSEETSNGDATTTES
PICPA_Rap1A 294 --LPQGIRGHYSAQDDYNLCLSV-----------------QPFIESIDETTGQEFFK-----------------------
PACTA-Rap1A 195 --LPKTLKSSFTAEDDYNLTKEI-------------------LSNTELSLDKDNNEF-----------------------
HANPO_Rap1B 210 --DIKKKTTNFSAWEDFEMCRTI-------------------YMKLRPTT-DEKGFE-----------------------
DEKBR_Rap1B 249 --LIKGRTTSFTAADDFALTKVI-------------------YQKLNPSE-KPNGFE-----------------------
CANAR_Rap1B 222 --DLRRKANGFTAVEDFLLCKDI-------------------YLNLNPIA-DEHGFE-----------------------
PICME_Rap1B 287 --QITSKLTPYTAEDDLLLCKTI-------------------YLKLDIVT-DDKGFE-----------------------
PICKU_Rap1B 291 --KVSNKLIPYNAEDDMILAKTV-------------------YQHIDFTV-DEQGFE-----------------------
CANBO_Rap1B 389 --TIKNKTTPFTATEDFLACKAM-------------------FTKLKPTL-DEKGFD-----------------------
PICPA_Rap1B 235 STLPGRVR--YSAKDDFQLCSQI-----------------LKYNVRESNINDVNDED-----------------------
PACTA_Rap1B 295 --LTINRSTPFTADDDYILAKKI--------------------TESAMITTNKEGKE-----------------------

**Myb2**

SACCE 480 --------LITDEDTPTAIARRNMTMDPNHVPGSEPNFAAYRTQSRRGPIARE--FFKHFAEEHAAHTENAWRDRFRKFL
SACCA 462 --------LISNEDSPTAIARRNMTMDPNHVPGNEPSFNDFRVNDRRGPVARE--FFKSFADANVSHSENAWRDRFRKFL
KLULA 333 --------LIAEDDEPNIVAKRQLVMNTEIDPSEVPSFEKYTVNDRRGPLSRE--FFKLFALEVPTHSENAWRDRFRKFI
CANAL 268 ---------------------------SIGLRYS---------------------FFDGMYRKYRNHTLQSWRDRFRKYI
CANMA 314 ---------------------------NINYT-----------------------FFSNLYRKRSSHTLNSWRDRYRKYV
DEBHA 297 -----------------GKPKPFDLYQEFTAPVS---------------------FFAMMAKNYPSHSYHSWRDRYRKFV
HANPO_Rap1A 320 ---------------------------GNSIPES---------------------VFTELVAKFPRHSAMSWRDRYRKFA
DEKBR_Rap1A 437 ---------------------------ESTVPEI---------------------VFKQMAEKYPRHSIMSWRDRYRKFA
CANAR_Rap1A 374 ---------------------------SENIPES---------------------VFLRLVEKSPRHTAMSWRDRYRKFA
PICME_Rap1A 507 ---------------------------VSQIPEA---------------------VFQELHKLNPRHSTMSWRDRYRKFA
PICKU_Rap1A 489 ---------------------------ISQIPEA---------------------VFQELNERNPRHSAMSWRDRYRKFA
CANBO_Rap1A 662 SKDSFDAAATAAAAAAAASTLTSALKAAATIPSVSSVLAAAGRKRRSNPNAIPESLFQELKDVNPRHSAMSWRDRYRKFA
PICPA_Rap1A 332 -------------------------------PLKG--------------------VFDDLESRFPHHTKTSWRDRFRKFA
PACTA-Rap1A 231 ---------------------------VLQYNIKK--------------------FYEYMQEKYSNHTANAWRDRYRKFL
HANPO_Rap1B 245 ---------------------------IINFPTG---------------------FFDNYASSHPRHTSESWRQRYKNFL
DEKBR_Rap1B 284 ---------------------------TVLFPTN---------------------FFDKFCHIFVQHTSESWRQRFKNYL
CANAR_Rap1B 257 ---------------------------SMNFTTG---------------------FFDLYHDAYPQHTAESWRQRYKNFL
PICME_Rap1B 322 ---------------------------SVVFPTN---------------------FFDKFAIVYDTHTPESWRQRYKNYL
PICKU_Rap1B 326 ---------------------------IMVFPTN---------------------FYDKFASVYDNHPAESWRQRLKNYI
CANBO_Rap1B 424 ---------------------------KLNFPTG---------------------YFDQYSKENPNHTPESWRQRYKNFI
PICPA_Rap1B 273 ---------------------------LKVNSS----------------------IFQELADSFPHHSKKSWSERYAKWV
PACTA_Rap1B 330 ---------------------------IVTWTTSK--------------------FFDNLAGTYPDHSKLSWRDRYRKFI

**Myb2**

SACCE 550 L-AYGIDDYISYYEAEKAQNREPEPMK-----NLTNRPK-------------RPGVPTPGNYNSAAKRARNYSSQRNV--
SACCA 532 L-TFGVDHYIEYFEQETNAGRKPEPMK-----NLTNRPR-------------RKAGITPGNYNSAIKRQRAYSISKAV--
KLULA 403 L-PYGIDSYISYYEKCMEEGIEPESIK-----NMTNRPK--------------REGPSPGNYNTTLKKSKRSTEGGTQ--
CANAL 300 RDKSDLVDYKEYYENCEKVGMAPRCLT-----------------------------------------------------
CANMA 344 D-EDTIPQYIEYYENCMELGEEPQCLT-----------------------------------------------------
DEBHA 339 R-QYGAQKYIDDYNRSIQNGETPEQMK-----NFTGKKA------GLTRGFKELIGSSYNDNYRALPSNENAGDDGAI--
HANPO_Rap1A 352 S-KYGLEEYMKYYEDCLAKNITPQPMK-----NLSSRAK------------------------DSKDVKRMKLDDKND--
DEKBR_Rap1A 469 S-VYGLEKYVKYYDDCIAAGKKPAPMR-----NLSSRAQ----------SRKRERDSLSVKSEDQNANKKPKIEDSKR--
CANAR_Rap1A 406 S-RYGIDEYCKYYEDCLAQNIEPVPMK-----NLSSRSK----------EATEARASKKSKISNNVSKSSSFIEDFDN--
PICME_Rap1A 539 A-KFGLRKYIAYYEECQQRDIEPEPMK-----NMSSRNDRKDYKVDVFGNESEERPSKRVKHEEDKILAKAKTVEDGKTF
PICKU_Rap1A 521 A-KFGIRRYIEYYEDCLKKNISPEPMK-----NMSSRTDRKDYKVDVFDNETKRPSTANIKPSKRIKLGTNKNTPKGE--
CANBO_Rap1A 742 S-QYGLDKYVAYYEECLKNGEAPQPMK-----NLSSRAN-------------RSDGSRKRKREEAEAAAAAAAATASSGV
PICPA_Rap1A 361 S-KYGVRQYIAYYEKTVELNGVPNPMT-----NFTSKAS-------------IEKFRERRGTSRNNGLPGPVGVEAVS--
PACTA-Rap1A 264 S-KYGITKYSHYYDECIYSGIEPESIK-----NFTSKAA------------LKRERENSTTHHDDNSTSNKKRRSNEQ--
HANPO_Rap1B 277 S-LFGIANYLKYCIMQIRQDKEPQPANSANKDWLNARKH-------------LKRASGPKLYFPNIPEGNEFLDANLE--
DEKBR_Rap1B 316 V-IFGIKNYLKYYIVQKKQGLDPQPSNLANKEWMRARRE------------LRKADNALRLYFPNIPMGNKFIDDNID--
CANAR_Rap1B 289 S-VFGIANYLKYYIVEYRAHRAPFPANLANKEWMQARKH------------QKRTGEGPLLYFPHVPTDNDLLKAEYL--
PICME_Rap1B 354 S-IFGIANYIKYYIMEVKNDEEPLPANIANKEWLQARKH-------------IKKTDCPRLYFPNIPQENEFIDENLY--
PICKU_Rap1B 358 I-PFGIVNYLKYYILEMKQGRKPLPTHKANKEWLQARKH-------------IRKTDCPRLYFPNVPLENDIIDENLQYL
CANBO_Rap1B 456 I-LFGIANYIKYYISTVKKNCEPLPVNLANKGWIVQRAA------------WKKNNTDEPLVYEEYDISDSDLLNELKTF
PICPA_Rap1B 304 S-AYGVGSYYRYYQARLENHEVPEPVS-----NLVLEKG-------------KYAASTALRQVRNIDSDDEVLHDVLV--
PACTA_Rap1B 363 S-VYGIQKYLNYYEKCEEADIEPVAVNVLLKETRKDRRH-------------EHPGDDVFQSARASPIPESSAKRRAT--
SACCE 609 -------------QPTANAASANAAAAAAAAASNSYAIPENELLDEDTMNFISSLKNDL---------------------
SACCA 591 ----HDQNSNISNAAVVAAANAASGDNTDTHSTPSYPIPENELLDEETMNFISNLKNDL---------------------
KLULA 461 --------------------------------QSTDTSLNDFFLESDAFDLIDGIRRDL---------------------
CANAL 327 --------------------------------------------------------------------------------
CANMA 370 --------------------------------------------------------------------------------
DEBHA 405 ----------------------------------------DEKIKQIRSSNYANAAENA---------------------
HANPO_Rap1A 400 ------------------------------------MVPSLDRIEALARVSVERKETEE---------------------
DEKBR_Rap1A 531 ----------EGESNVSVSVSDLGVAQIQELARASVQKSADEEMKDGASESSKKQETAA---------------------
CANAR_Rap1A 468 --------------------------------ESDKKSTNLERIEALARVSVERKETLD---------------------
PICME_Rap1A 613 EGSDQLTESDTAYMALSKAKGKLGTETFADGSLSETSAAEGALEALANVSAEQTKANEI---------------------
PICKU_Rap1A 593 ---HTSYHLKDSGQSDAKILEKPNTSVLADPLTNNDESAAVGALKELAKVSFQQNGTAG---------------------
CANBO_Rap1A 803 SSSTDDKSHETAAPKDKKSKTSASTSTATTTTTEDEKKDKKSKKSKRKATDEISDDVEA---------------------
PICPA_Rap1A 420 ------------------------------------------SLDQISPLVTSNSNSAA---------------------
PACTA-Rap1A 324 -------------------------------------------------EIITNNEEIL---------------------
HANPO_Rap1B 341 ---------------------TMEASDLVEPEFVPAPRPENQFLRPTPQEQIEQSIAMA---------------------
DEKBR_Rap1B 381 --------------------------------MIEVPELEGKNLEVFTPVIISSNSNDS---------------------
CANAR_Rap1B 354 -----------------------------ESIPVPEPRPKNDRILATTASMFLEPKPML---------------------
PICME_Rap1B 418 --------------------------------YIALPAYDTKIFEFDNPFRNIKKEES----------------------
PICKU_Rap1B 424 NIKEANKEFNYTNPFRELNKRRSEEIDETNPSIRTADTTEVGIDDDNNHDNGTKHANSS---------------------
CANBO_Rap1B 523 EPPQTYPESEAINNARKTLKNEARIALKLLNNNQTHSATTDENEESEEMEEIENSEEDEEVGQAKKGKKQDHKQREQNVE
PICPA_Rap1B 363 --------------------------------------------------------------------------------
PACTA_Rap1B 427 --------------------------------LDGLQSASKVLKTSNPANSNDDRATDV---------------------


SACCE 655 ----------SN-----------------------------------------------------------ISNSLPFEY
SACCA 646 ----------SKLESNNSG----------------------------------------------------NNNNLPFEY
KLULA 488 ----------HAAETQ-------------------------------------------------------QEEQARSLY
CANAL 327 --------------------------------------------------------------------------------
CANMA 370 --------------------------------------------------------------------------------
DEBHA 424 ----------GEHEIQ----------------------------------------------------------EGEGES
HANPO_Rap1A 423 ----------NAAKKEDSPEAKGEKDKRLDDGS--------------------------------------ASANLFEDA
DEKBR_Rap1A 580 ----------DSALSQ----------------------------------------------------------SANLFE
CANAR_Rap1A 495 ----------NETTTTTTTTSVDNEKVALKKVEEGKKDDGS------------------------------DGANLFADA
PICME_Rap1A 672 ----------SSNDSANVQDNLKTDETEAADTKTLSAGKKSNARSSKAVDGVSYKVSQTVESVSND-----DGANLFAAA
PICKU_Rap1A 649 ----------DENLPKEKPSTRTNDGEENEDTNEVHGSSREDADD--------------------------DNANLFAHA
CANBO_Rap1A 862 ----------KKQRTDASNQADELDELARISVERQNDESKQQHGYLNDAEEARAVASLASAVSADTTRIAREAAAVVEAA
PICPA_Rap1A 437 ----------AAAAAA-------------------------------------------------------AAVAASASA
PACTA-Rap1A 334 ----------TSEEI--------------------------------------------------------SRPITFQDN
HANPO_Rap1B 379 ----------AQQANQDLVTAFKNFRQEEQ-----------------------------------------DGPPAKRAK
DEKBR_Rap1B 408 ----------DEEPVRKK--------------------------------------------------------QHTSNN
CANAR_Rap1B 384 ----------TQEQNIAAAAQRQQAVREADDHDDQLHPA--------------------------------KRSKITPQD
PICME_Rap1B 444 ----------DGFSPEPIP----------------------------------------------------SSSAGTTEM
PICKU_Rap1B 483 ----------KNHSKSDTDTEKHKQAEMSKEIPGESSSEENKIKRQKVREVDSPEESGITFHIDENKQKPVENEQEKHQQ
CANBO_Rap1B 603 GLVEPADLVDDASSSSLSPATSTEANPQNDDNNFVIHSEDVSDEELAAAAAAAAAAASAAVYEAARASKRALKASETPRS
PICPA_Rap1B 363 --------------------------------------------------------------------------NAKRRK
PACTA_Rap1B 454 ----------SPSKVGHKRA---------------------------------------------------ETNDLDSKL

**RCT**

SACCE 666 PHEIAEAIRSDFSNEDIYD---------------------------------------------NIDPDTISFPPKIATT
SACCA 664 SPEIAEAIRNDFDNEGKEF--------------------------------------------DNIDPDTIKFPPEIATI
KLULA 503 ADDVAERIRHQVALEHEEY--------------------------------------------DNYDFDSIPFPPKLADN
CANAL 327 --------------------------------------------------------------------------------
CANMA 370 --------------------------------------------------------------------------------
DEBHA 436 NNDIESVIESQSQAESQPQ-----------------------------------GKGGSLLDSQSTSQMWSDFDIKYLPE
HANPO_Rap1A 455 NEDEIKALNDDLSKFDALP----------------------------------------------SIETEGNFSFEELKK
DEKBR_Rap1A 592 DADEMKALNIDFTGSLSSH-----------------------------------------------NGLTGTITLSSLEK
CANAR_Rap1A 535 NEEEIKALNENLPELPPLE-----------------------------QEQEQEHEHEPVTEVEAEPVTDAILSFEELKR
PICME_Rap1A 737 TEDEMKQYDIALADSQLEE----SNTNTDKKDAAEIMSEHVSPEISESTAAHLGEEGDNDLELVDVKADDGLMDFRQLID
PICKU_Rap1A 693 TVDEMKQYDIGMDTSGIEN--DEESLKKKSVVSLSRVAETSRDESRDEENETDTNSVGNDAELVNVKADDGLMDFRQLME
CANBO_Rap1A 932 TGSTETDIDGVVSDETKDD------------------KSDPNAMSGANIFEIMEEAHKALEEHLKSENAAQKALESVDSL
PICPA_Rap1A 452 SSAPNTSTTNFFEQENIAQ------------------------------------VLSAHNNEQSIAEVIESAQNVNTHE
PACTA-Rap1A 348 KDDENHDNTNYFAQQALSL-----------------------------------------------------GDEKIQED
HANPO_Rap1B 408 RDQRSSSANIDPAIEGMQP-------------------------------------PLEFVDEPTTYFSDPRFALSLKNE
DEKBR_Rap1B 422 QPHQIESYDPELKEEPKTK----------------------------------PEQIIAFVDVPTTSFREKTFVRSYKKY
CANAR_Rap1B 422 ASNSNLTIDPQIGELHYNE-------------------------------------TPAFVDDVTSSFLELTFSRSRVNY
PICME_Rap1B 462 VNDTEKAIGVATAGSDYNG----------------------------------------FVDEPTTEFIEVAFKKSYRKY
PICKU_Rap1B 553 NQQRQQPYNNLFQEGDRET----------NQENSHAFKMEKQSEVRTRPNEDSDDVPSVFIDEPTYKFTDSTFAKSFKKL
CANBO_Rap1B 683 AVNEVSSKNREEVTSPSKKTTTSLSQRKTKQRSTPDTNLPPKKKHKADSNKDQHDLIEEFKDLPTTFFLEPTFAKSFQKF
PICPA_Rap1B 369 HSSSVIPVSSTTCKSDLSR----------------------------------------------------VFLQSLQRD
PACTA_Rap1B 473 DKETENDVNDSKNQSEKLQ--------------------------------EQQAIVEGEEEVPDVSDDSDWQESEKVKE

**RCT**

SACCE 701 DLFLPL----FFHFGSTRQFMDKLHEVISGDYEP---SQAEKLVQDLCDET---GIRKNFSTSILTCLSGDLMVFPRYFL
SACCA 700 DLFLPI----FFQFGSTRNFLEKVENVIKRDYEP---SQAEKLVEDLCDEA---GVRRGFSTSILTALSGDLMIFPRYFL
KLULA 539 DDFFDH---TFFNFRSTKEFIQKLEEIITREYDE---SQADVLVHDLDVEC---GIRKKYATSVLTALTGDISLFPRYIL
CANAL 327 ------------------------------------------------------RIAP----------------------
CANMA 370 --------------------------------------------------------------------------------
DEBHA 481 GITLEHLLNKNFFKISPNEISTRVETILNDIEL----AAPAVLLKSFNEI----GINDVFTAHLIMAATGDIVNMQVFID
HANPO_Rap1A 489 EDPEPL---KNKSLINVEETCAQIEQIFGSFGSSI--TTSFELFKVMNEKV---GLSMGWLTYWFDCSCGHLNLFMAAIL
DEKBR_Rap1A 625 AEPEPI---EDREKVNVEETIKDIEDLFGDFGSDI--RSSEQLCEAIHKKT---GISAAWLSYWFDCSCGMINVFLDVVQ
CANAR_Rap1A 586 DEPEPI---KDKSELNIEKTCNEIESIVSNFGSNL--TTSFDLFKVISEES---GLSLRWLNYWFDCSCGHLTVFMDAIL
PICME_Rap1A 813 IDPEPL---KHRGEIDLSTMISNIRECFRNFGDG---NTPYELFKDISDQT---GISMLWLNYWFDCSCGMLGTFIQAII
PICKU_Rap1A 771 IDPEPL---KQRSEIDLETMITNIHECFRNFGDG---NTPYELFKDISDNT---GISMLWLNYWFDCSCGMLGTFIQAII
CANBO_Rap1A 994 TKISPI---DNRKDKDTDELIKTVSESFEKAKTEK--MSKSEIFQLLFEKA---SVNSKWSSHWYECSSGDLSVMLSAIK
PICPA_Rap1A 496 SEPIAD---HVRKNLTDDELLDKMDDILSSRSL----GGLDDLIKILHTEL---GFAHRYTEFLFTSCSGDVIFFRPLVE
PACTA-Rap1A 375 EQLIDK---NILNSMSNTDFLAKLSFIIENISKKGEDIDTDELLKILKLEL---GISILFSSNIIFHVSGDLSKFQNFLD
HANPO_Rap1B 451 GPPIDL---ADVLP-RKESFLEQLDKTLDPQVT----VTTKTLFSQLASL----GIKEYYIIFLFHRCNSRRNLVLDCIK
DEKBR_Rap1B 468 GEPMDL----KSIVASKPAFYAKLDSVFSQGS-----LSPKQLSFELRKI----GIKEYYTVFLMHRCNSNRQLVKKSIC
CANAR_Rap1B 465 GPPMDL----QAIASYKEQFDKMLYTILDPSTA----MSPKNLSKELANL----GIQEYYSVFLMLRCNSKKSLVLECIK
PICME_Rap1B 502 GPPINL-----ASVSDKKALITTFKKIFSVQGEK---LAPRELSKRLLEE----GVQEYYTVFLIYRCNSIKSLVQESLL
PICKU_Rap1B 623 GPPMEL------INVNKDDLIDKINQIFEK-------HGPKILPRLLSKELSAAGINEYYTVFLIYRCNLVRDLVLKSLI
CANBO_Rap1B 763 GPPVVL----TEIKKHRDEFIHKIYNTIDPSNT----TTPKEVADKLGTI----GIKEYYTVFLMHRCNSNRKLVAKCIQ
PICPA_Rap1B 397 SPPIDP---EKIEKIGKDAYTKLLKDVLAEVKIF---DSHQEWKSTFKNAL---GVKDYYLTFLLDVTSGNLKLIVQIIS
PACTA_Rap1B 521 DKFISS---EILSSSGPKAFISRLSSLIDSSTT----DNGSEICQLFEKEL---GISTDFSETVLFRVSGDLSKFTEYVK

**RCT**

SACCE 771 NMF---------K-DNVNPPPNVPGIWTHDDDESLKSN---DQEQIRKLVKKHGTGRMEMRKRFFEKDLL-------
SACCA 770 NMF---------K-NNVNPPLNVPGIWTHEDDAMLSSN---DSEDLRHLEKKHGAARIAIRRRFVERDLV-------
KLULA 610 TSF---------K-YGVHLPQNVPGIWTKEDDVILRSK---NPDGMKMLEKKHGIARMQMRIRFQENNLV-------
CANAL -----------------------------------------------------------------------------
CANMA 370 --------------------------------------------------------RVNK-----------------
DEBHA 553 QFLHLIPHLSEDKLYDGLQIEDTDGIWTSKLDNYLLSG---SKQDMKKLQAIQSPQSIEARRHFLEKFS--------
HANPO_Rap1A 561 NYI---------R-NDELILSNYPGFWTSEHDRMLK-----EEDKISDLIKLHGLESVTKRREALYGE---------
DEKBR_Rap1A 697 NYL---------K-TGELIMKNHAGFWTEDQDRMLL-----DESKLPDLYKLHGTESVNKRRAALVGQSSNVVS---
CANAR_Rap1A 658 NYI---------R-NDELILKDYSGFWTDEHDMMLK-----NDDKLNELIELHGFESVQKRKEALFGVD--------
PICME_Rap1A 884 NYL---------K-TGDLVMNDVSGFWTEKDDELLK-----VDPENKDLLHLHGKDSVTKRKAVLFRYV--------
PICKU_Rap1A 842 HYL---------E-TGELVMNNVSGFWTERDDELLR-----VDPENKELLKLHGKDSVTKRKAVLFSYVL-------
CANBO_Rap1A 1066 NYL----------LNDELELKDTKGYWTLEQDELLKKG---NEDDIKELVKIHGADNVEKRKSKFED----------
PICPA_Rap1A 566 HFL----------LTGEWELENTRGIWTGRQDEMLRAS---NLDDLHKLIDLHGKERVETRRKAIKGE---------
PACTA-Rap1A 449 IYL---------K-TGQNPPVDYSGIFIKEYDDIIANNFDENKSPLLEMVKLHGKKKVRFRRKFLKSLSI-------
HANPO_Rap1B 519 NYL---------DTNGKELLVQKPGVWSNKALEWLGKN---DSHLNSLLELYHGEHDYRTQLENMRKIKQ-------
DEKBR_Rap1B 535 HFI---------ETNGHELLCIGPGAWSNKALSWLDKQ---NEHLNTLLKKYHGEENFESELRSLRRTKSISW----
CANAR_Rap1B 533 NYI---------ATDGAEYLARRPGIFSNKAVEWLRTR---DPSLLQILKEYHGEAEVEQQLKSVTKTNSNEWKNAK
PICME_Rap1B 570 NYV---------ETDGAELLVMKPGIWSNKCIEMFEKH---DPKLEKILKSYHGEKGYVKQARWLKTSKMDFVR---
PICKU_Rap1B 690 NYI---------RTDGKELLIIAPGVWSDKGMEYFEKK---DPRYDSLLISYHGEKAFKRQCKWRQKDKKQK-----
CANBO_Rap1B 831 NYL---------NTDGKELLAMKRGVWSNKAIEYLRSG---NSKLLSSLKKYHGAESFKIQSESLKRSKK-------
PICPA_Rap1B 468 NYL---------E-DNTQYLTNFPGVFLPRHDIILLANE--NKDEIDQLIKYHSLENVEKRRKYLF-----------
PACTA_Rap1B 591 IYL---------K-TGLNPPKGIVGIFSDEDDEILQNFHS-QPNKVKALVKLHGLKTIKFRRKFLKEHYS-------


**Supplementary figure S1.** Multiple alignment of Rap1 homologues from budding yeasts.

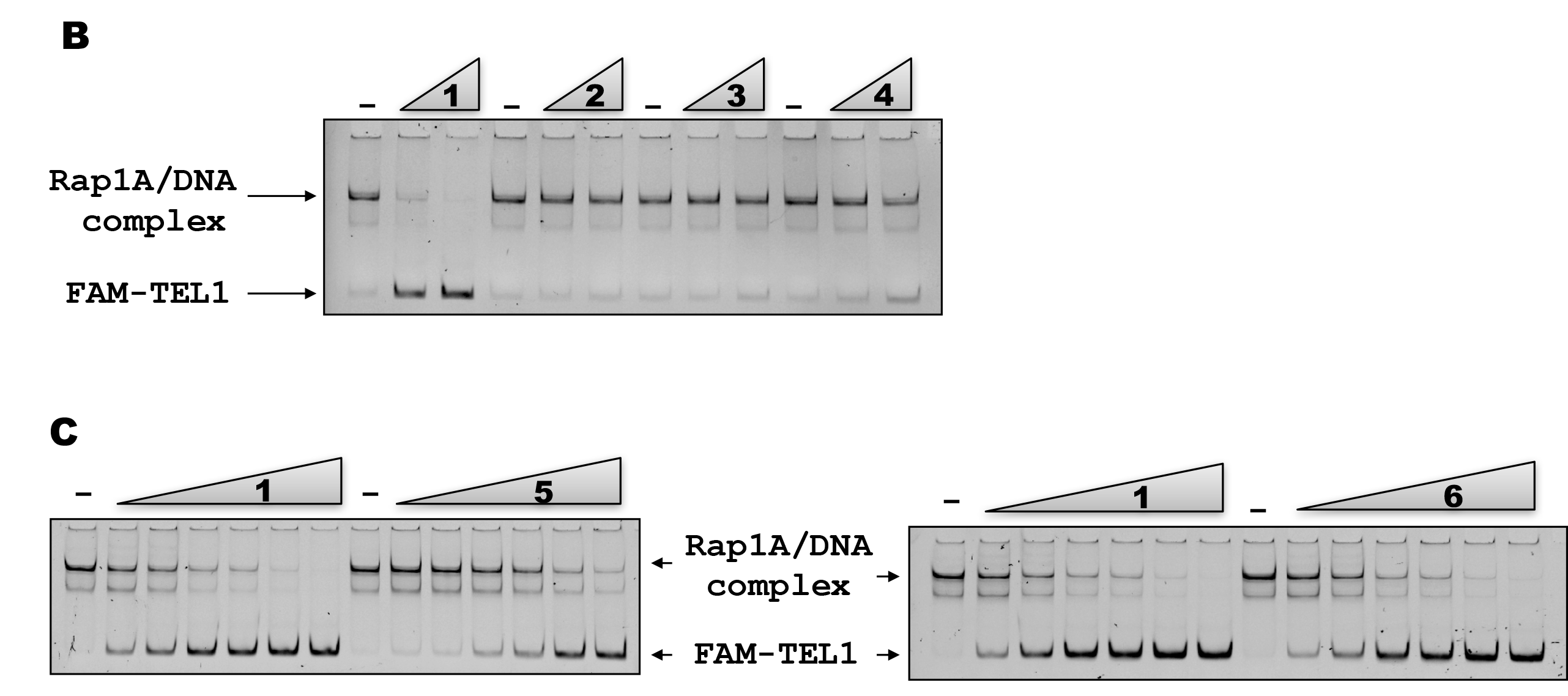


**Supplementary figure S2.** Localization of the high-affinity HpRap1A binding site within the right end of chromosome VII. (A) Schematic of the regions of the right end of chromosome VII used in the competition EMSA (upper panel). +1 – is the first base pair of the first telomere repeat. The 13-bp sequence within oligo #6 (or “VIIrAbs”), which is almost identical to the Rap1 binding site within telomeric repeats from *C. albicans* is underlined. (B and C) Competition EMSA. The 2 nM FAM-TEL1 was incubated with 40 nM HpRap1A in the presence of 0, 225 nM or 2250 nM of double-stranded oligonucleotides 1, 2, 3 or 4 (B) or 0, 22.5, 45, 225, 450, 2250 or 4500 nM of double-stranded oligonucleotides 1, 5 or 6 (C). The images in (B, C) are cropped from three different gels and delineated by black dividing lines. Full-length gels are shown in Supplementary Fig. S7.


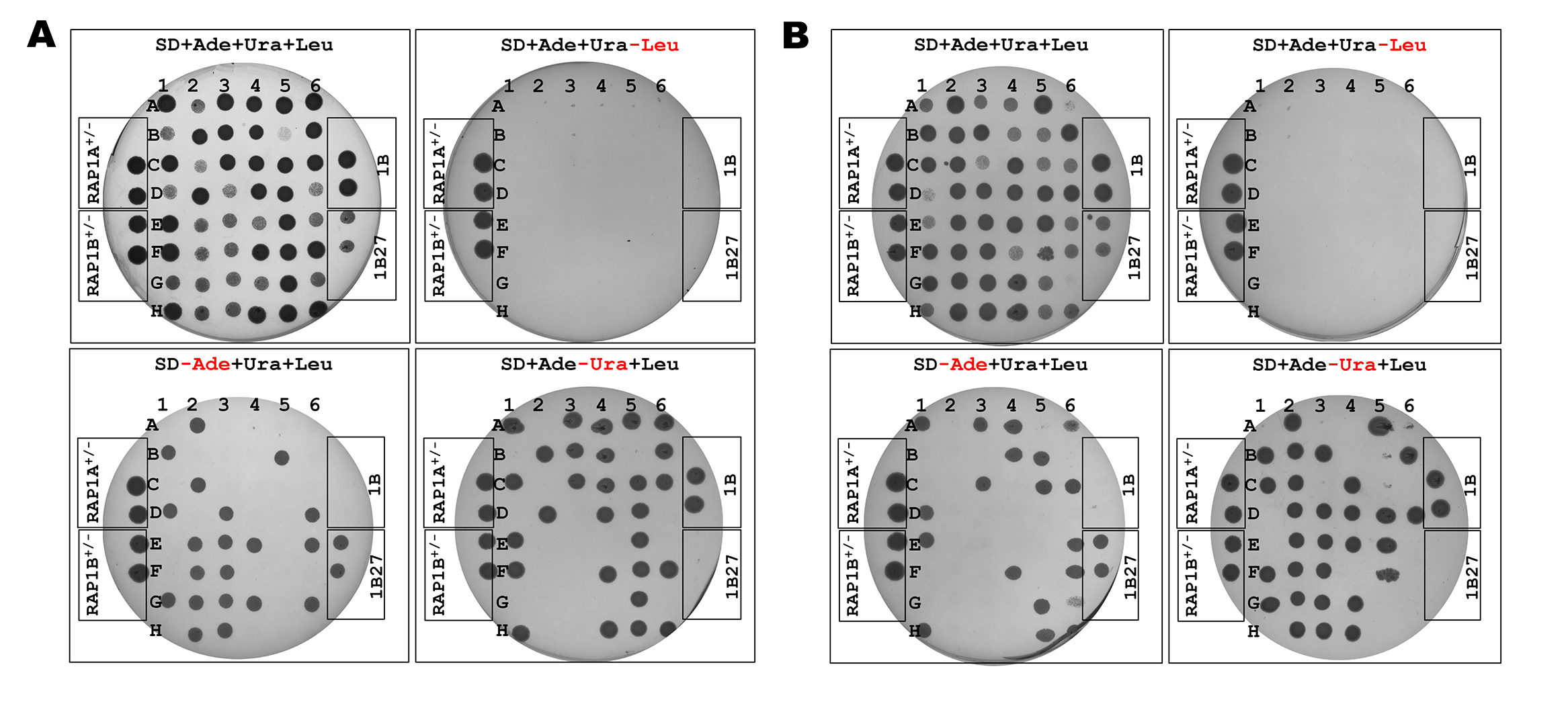

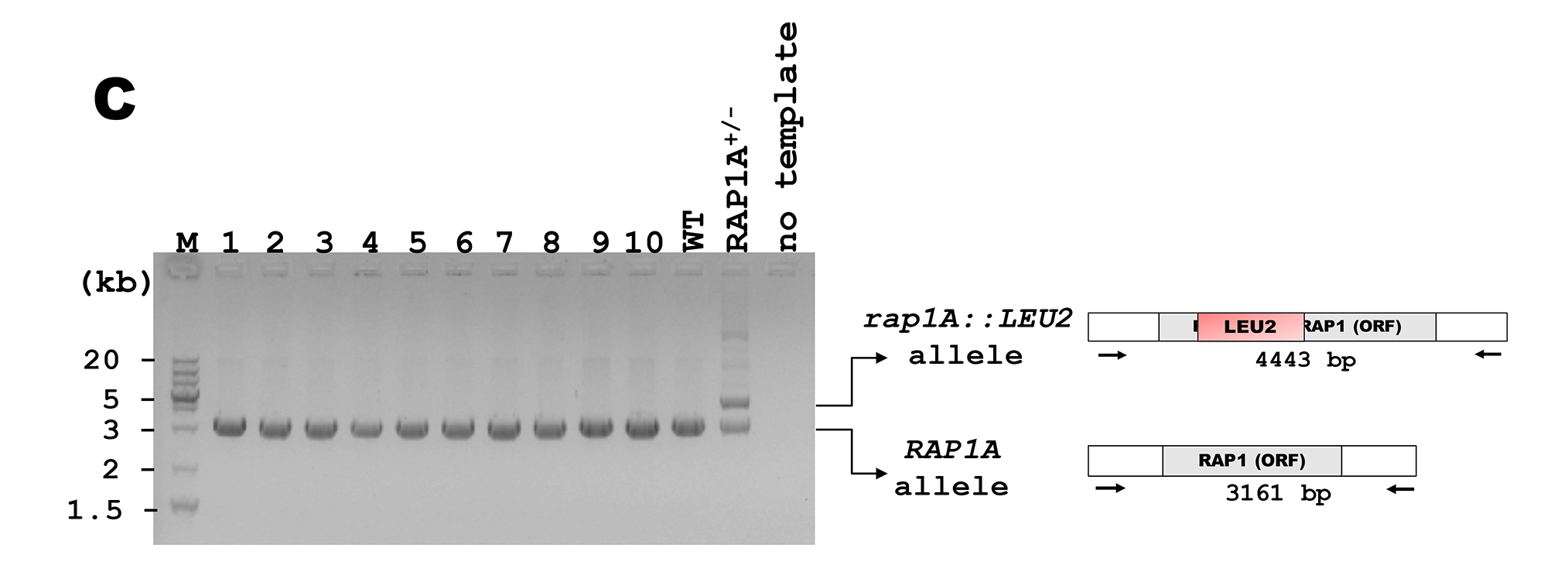

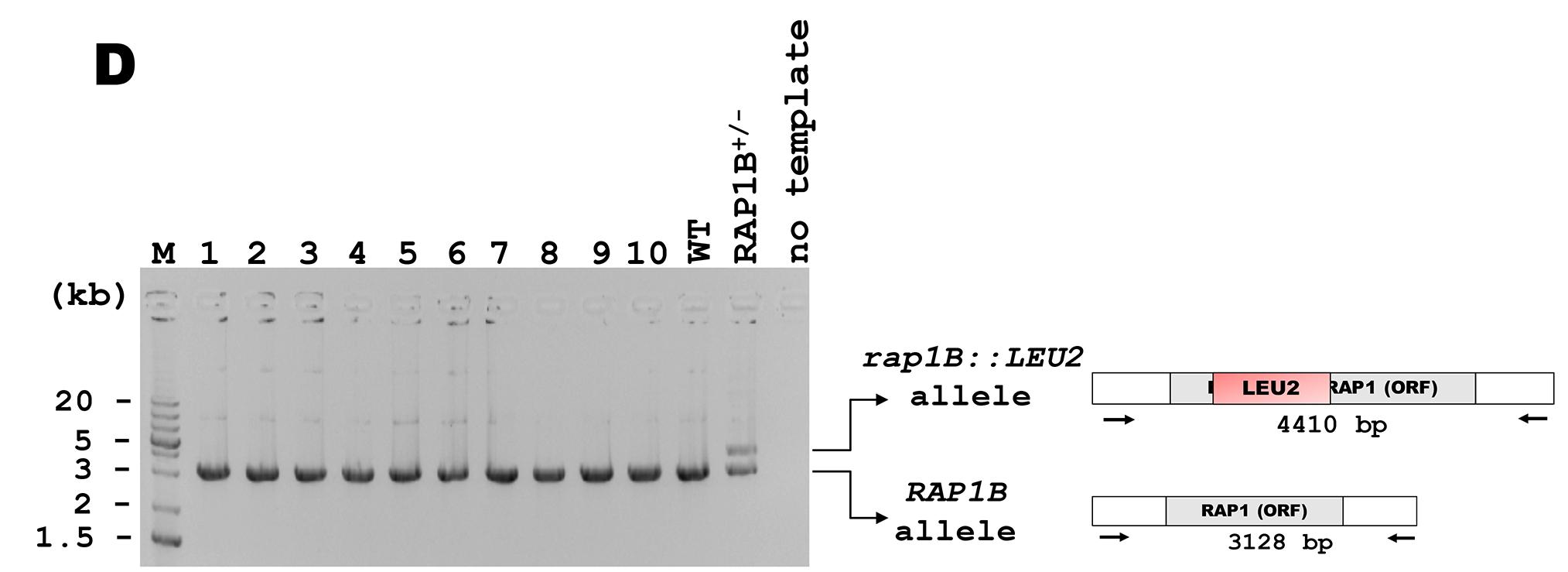


**Supplementary figure S3.** Results of the analysis of random spores derived from either heterozygous RAP1A^+/-^ (A) or RAP1B^+/-^ (B) strains. (A) After ether treatment and growth on YPD plate, 48 colonies derived from the heterozygous RAP1A^+/-^ strain (A1 – H6; each from a single spore) were randomly picked, resuspended in water, plated onto the indicated selective plates, grown for 4 days at 37 °C and photographed. Each plate also contained the parental RAP1A^+/-^ and RAP1B^+/-^ strains (phenotype: Ade^+^Ura^+^Leu^+^), 1B strain (phenotype: Ade^-^Ura^+^Leu^-^), and 1B27 strain (phenotype: Ade^+^Ura^-^Leu^-^). None of the spores displayed the Leu^+^ phenotype. 42% (20/48) displayed the Ade^+^Ura^-^ phenotype, whereas 58% (28/48) – Ade^-^Ura^+^ phenotype. Note that diploids generated from the 1B and 1B27 strains should produce spores with an Ade^+^Ura^-^ to Ade^-^Ura^+^ ratio of 50 to 50 (close to our experimental value). (B) Same as (A) only for the RAP1B^+/-^ strain. Among the spores, 40% (19/48) had the Ade^+^Ura^-^ phenotype, whereas 60% (29/48) – Ade^-^Ura^+^ phenotype. (C) PCR analysis of genomic DNA isolated from 10 colonies derived from heterozygous RAP1A^+/-^ (each from a single spore; not the same as in (A)). M – marker, “1-10” (10 haploid spores), RAP1A^+/-^ (parental heterozygous RAP1A^+/-^ strain), “no template” – PCR with no gDNA. Expected lengths of the PCR products are indicated in the schematic on the right. (D) same as (C) only for the RAP1B^+/-^ strain.

**Supplementary figure S4.** A photograph of a YPD plate with the indicated strains grown for 2 days at 37 °C.


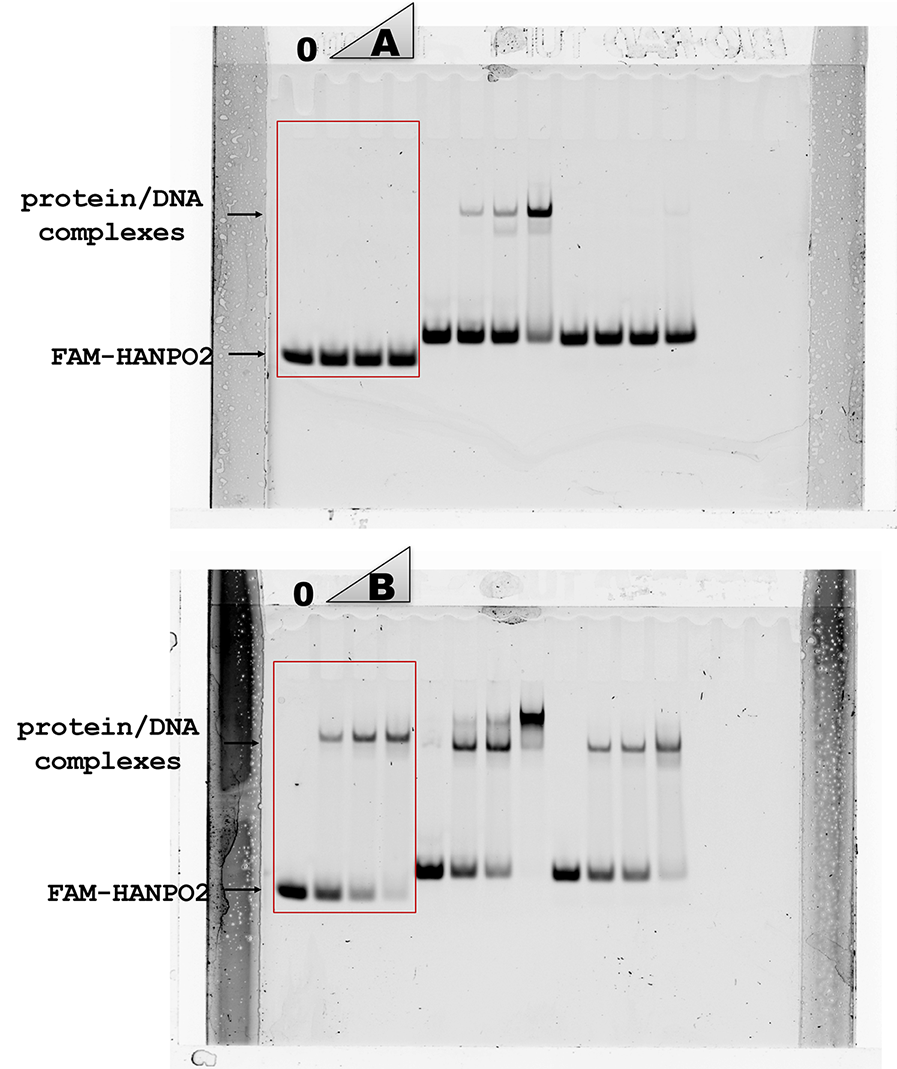


**Supplementary figure S5.** Full-length gels for Fig. 2C. Red squares show the areas that are displayed in Fig. 2C. The 50 nM FAM-HANPO2 oligo was incubated with an increasing amount of either HpRap1A (upper gel) or HpRap1B (lower gel) protein (concentration range: 0, 50, 100, 250 nM).


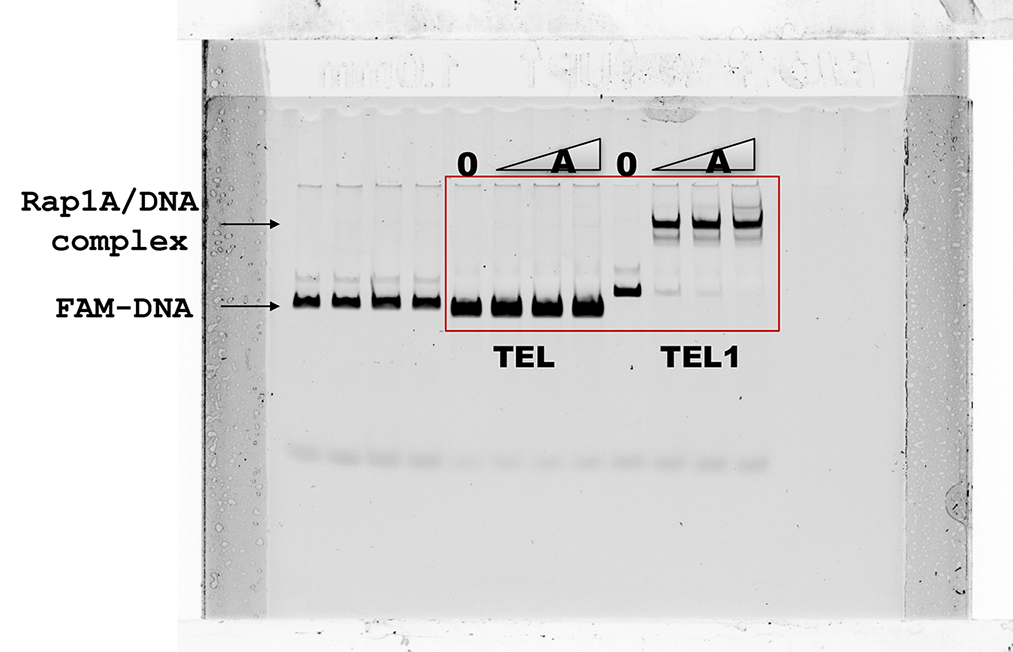


**Supplementary figure S6.** Full-length gel for Fig. 2D. The red square shows the area that is displayed in Fig. 2D. The 10 nM FAM-labeled genomic fragments (“TEL” or “TEL1”) were incubated with an increasing amount of HpRap1A (0, 10, 25, 50 nM).


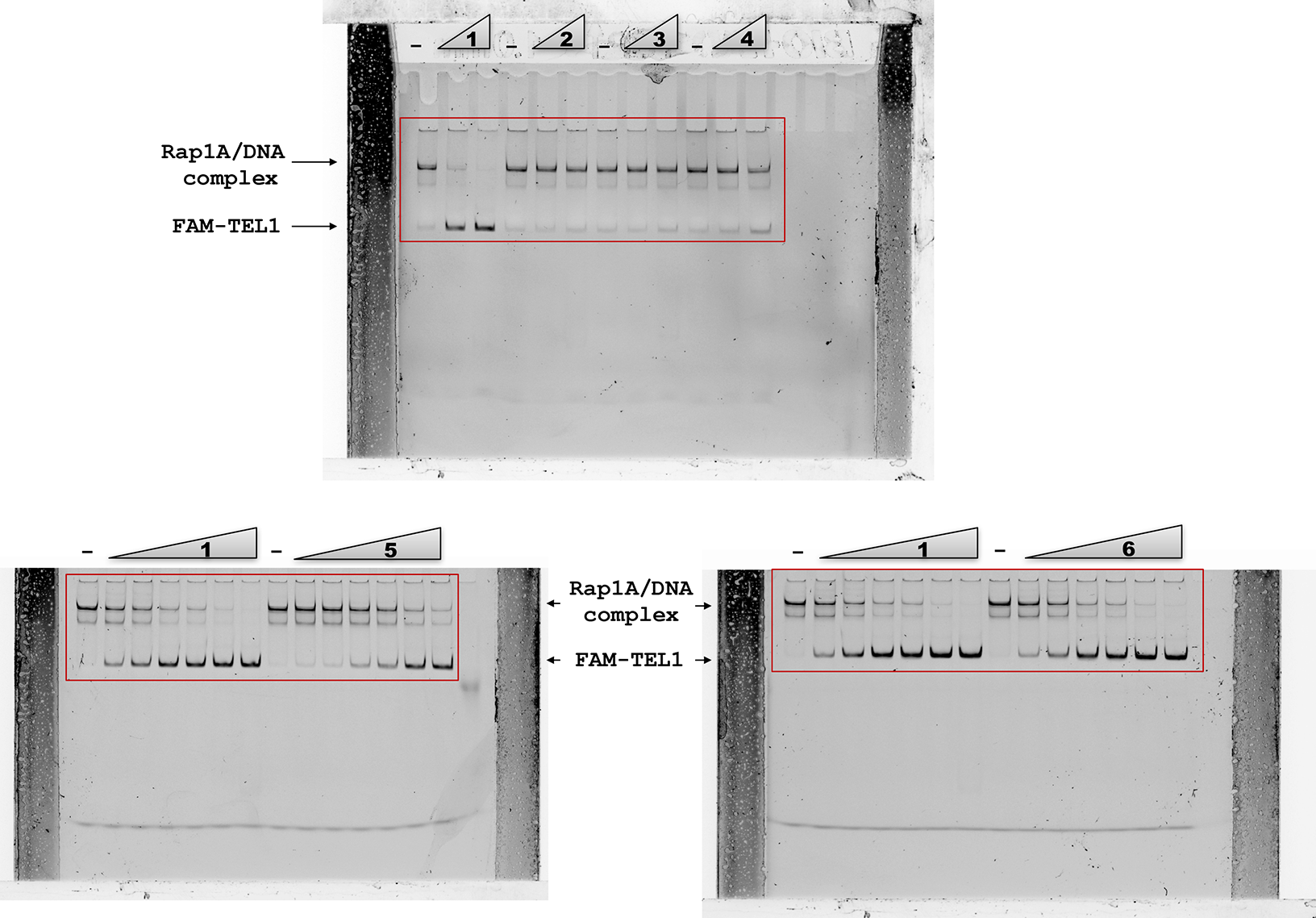


**Supplementary figure S7.** Full-length gels for Supplementary Fig. S2B and C. Red squares show the areas that are displayed in Supplementary Fig. S2B and C. The 2 nM FAM-TEL1 was incubated with 40 nM HpRap1A in the presence of 0, 225 nM or 2250 nM of double-stranded oligonucleotides 1, 2, 3 or 4 (upper gel) or 0, 22.5, 45, 225, 450, 2250 or 4500 nM of double-stranded oligonucleotides 1, 5 or 6 (lower gels).


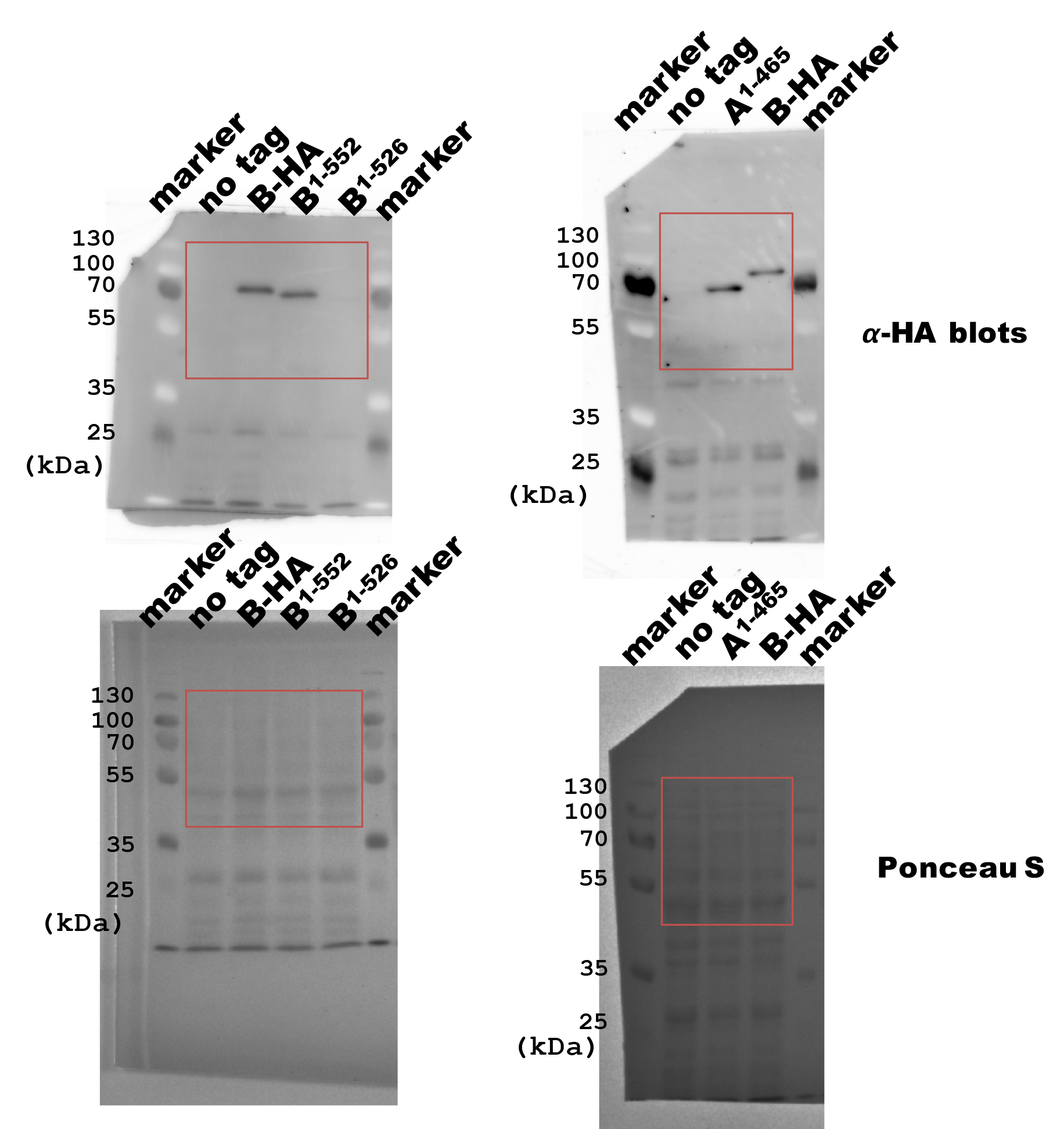


**Supplementary figure S8.** Full-length blots for Fig. 4C. Two upper panels – anti-HA Western blots of the total proteins isolated from the indicated strains. Two lower panels – Ponceau S-stained membranes. Red squares show the areas that are displayed in Fig. 4C.


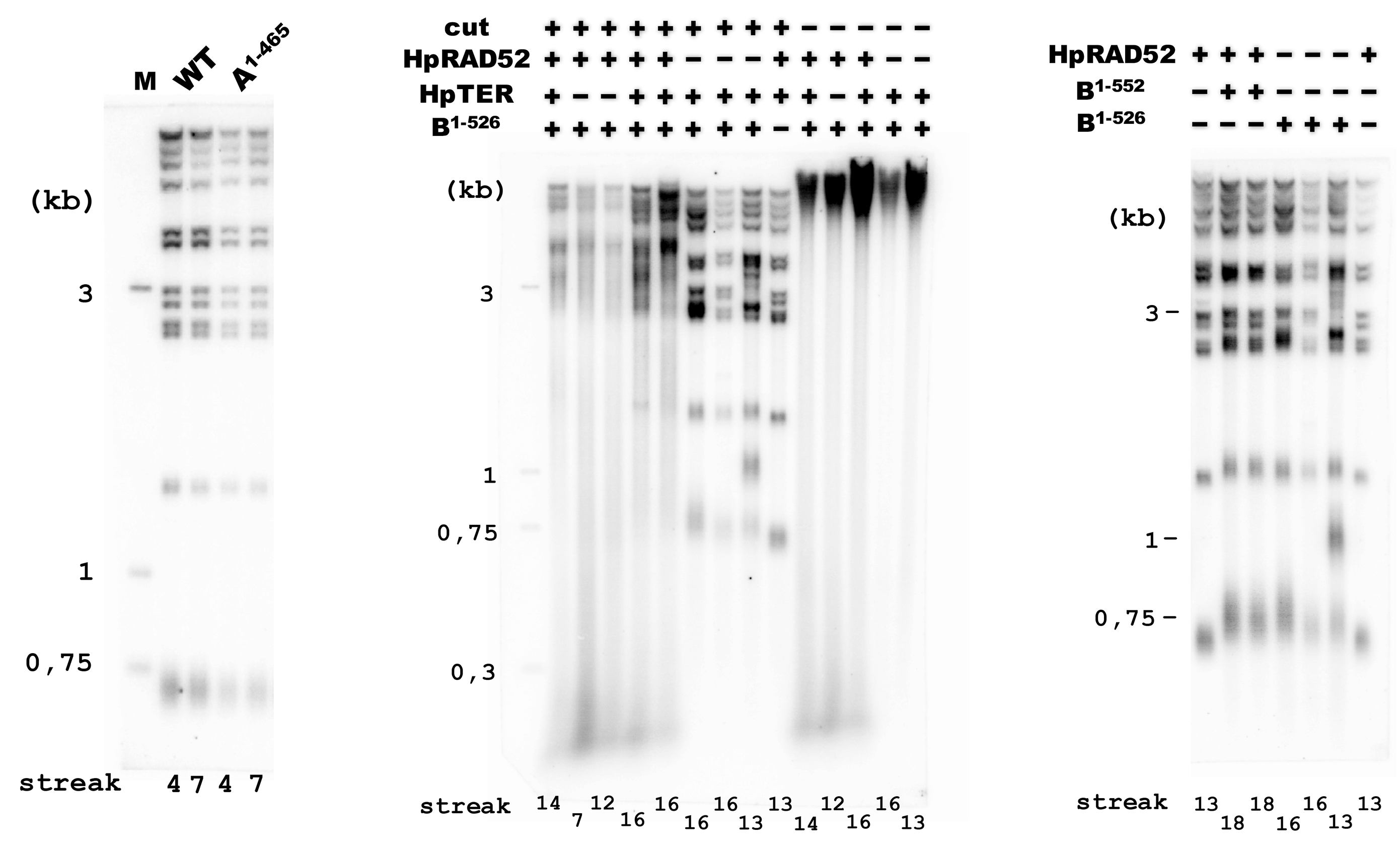


**Supplementary figure S9.** Original (no contrast adjustment) Southern blots for Fig. 4D (left panel), 4F (middle panel) and 4G (right panel).
